# Supplementary material for: Fungal community and taxa specialization to host and environment interactions in two temperate forests
Source: PLoS One. 2025 May 9;20(5):e0322440. doi: 10.1371/journal.pone.0322440 (PMC12063886; doi:10.1371/journal.pone.0322440)
Supplement: S1 File — (PDF) [file pone.0322440.s001.pdf]

Fungal community and taxa specialization to host and environment interactions in two temperate forests

Maria Soledad Benitez Ponce, Michelle H. Hersh, Lindsey Becker, Rytas Vilgalys, James S. Clark

S1 File. Supporting Figures S1A-S1J

Corresponding author:
Maria Soledad Benitez Ponce
e-mail: benitezponce.1@osu.edu

Figure S1A. Schematic of location of plant soil-feedback (PSF) plots within a research site. At each site adult trees were mapped into a forest grid. Adult tree and grid location were used to determine placement of PSF plots at specific distances to conspecific adults. In addition, depending on the site, location of gaps, were also considered.

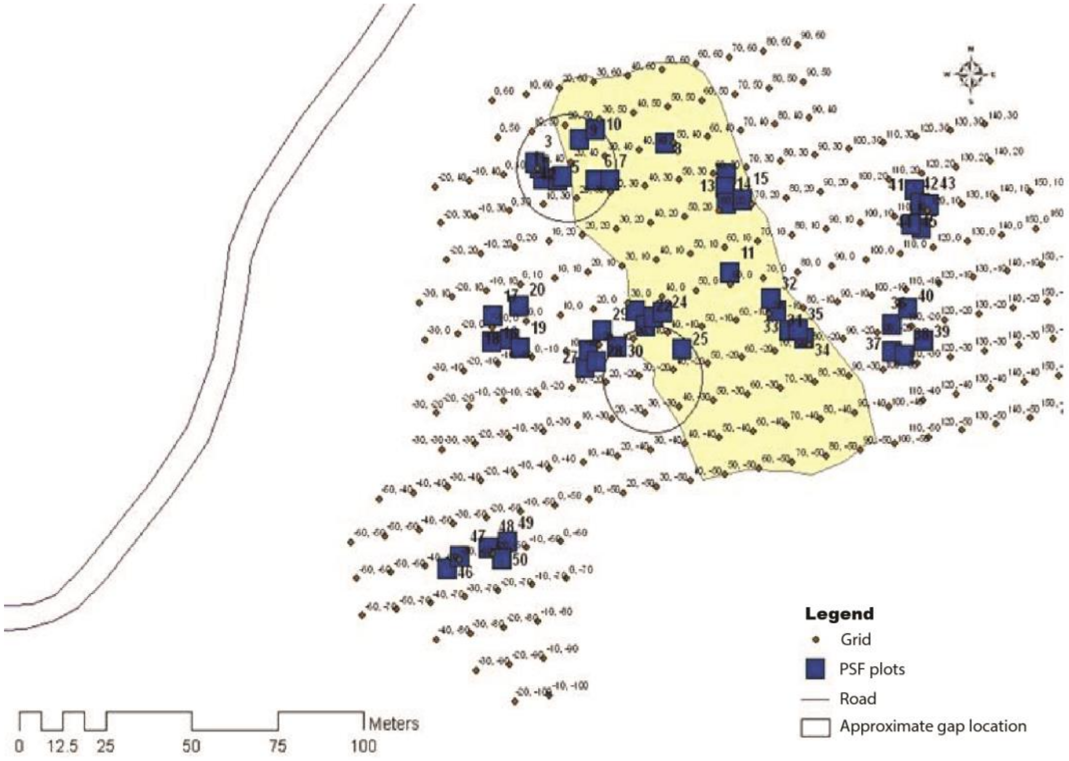

Figure S1B. Relative abundance of the ten most common OTUs recovered from each of the nine plant hosts studied. Bars represents summed relative abundance of individual OTU within sample analyzed per host species and disease status (A: asymptomatic; S: symptomatic). Relative abundance per sample was estimated as proportion of the number of reads per OTU in relation to the total read count per sample. OTUs are organized alphabetically, and the OTU Colletotrichum_1 was the most commonly recovered OTU, representing 8% of the sequences in the dataset.

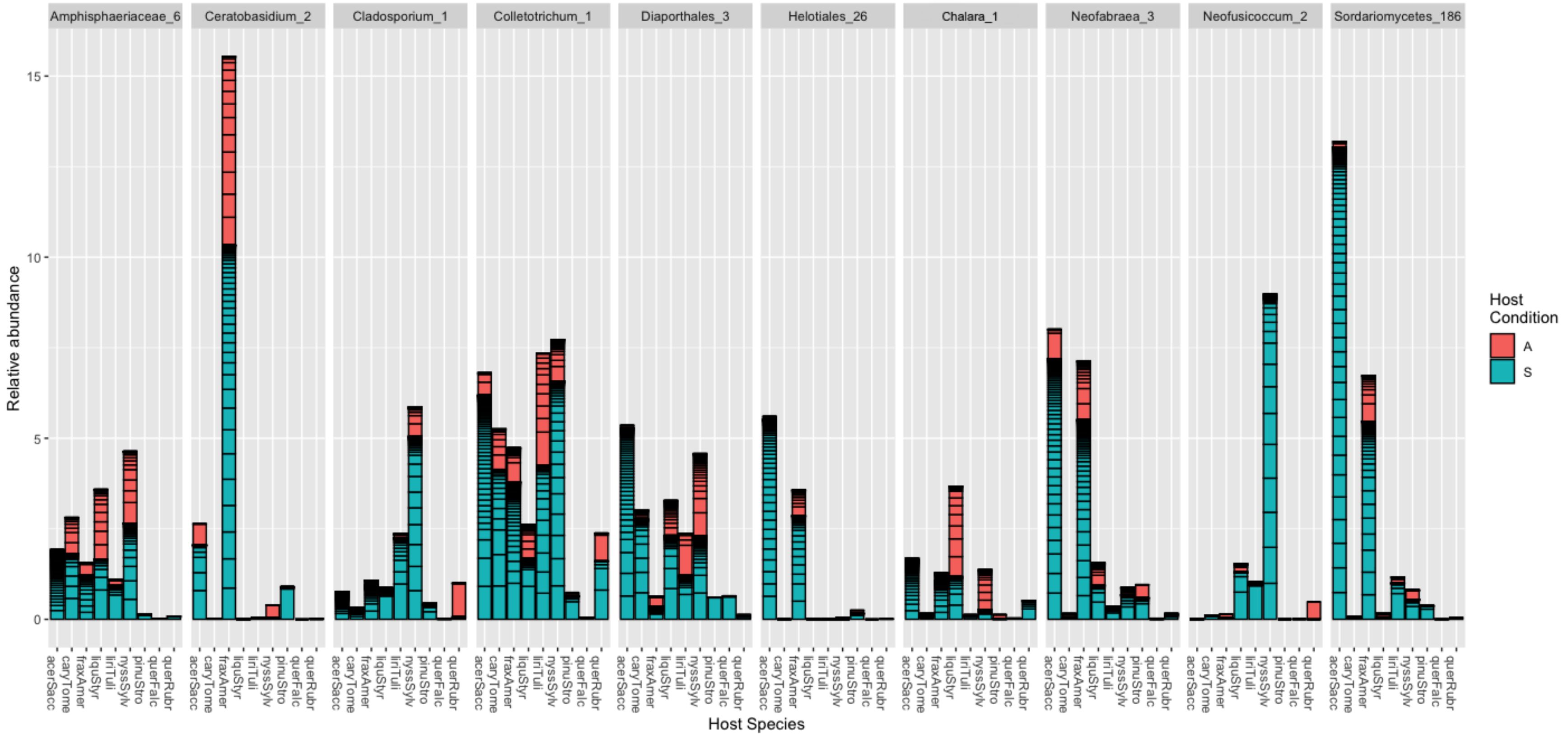

Figure S1C. Variation in alpha diversity metrics, Chao1 (this page) and Shannon (next page). Box plots represent distribution of diversity estimates within samples from different host species, graphed by sampling location (DFEW, DFHW, DFWS=Duke Forest Eno West, Hardwood and Warming Site, respectively; HFBW, HFST, HFWS=Harvard Forest Barre Woods, Simes Tract and Warming Site, respectively) and health status (A=asymptomatic; S=symptomatic).

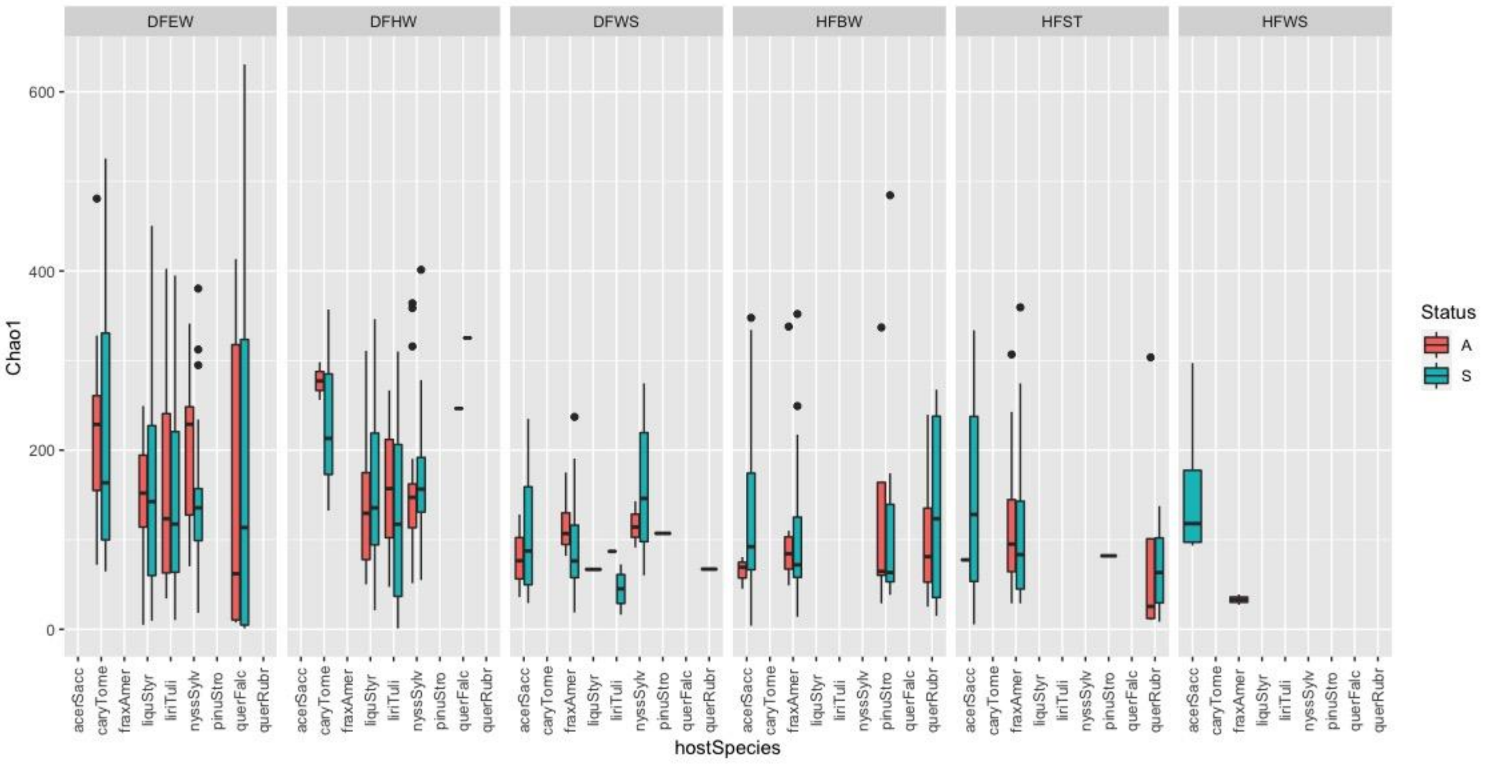

<u>Chao1</u>

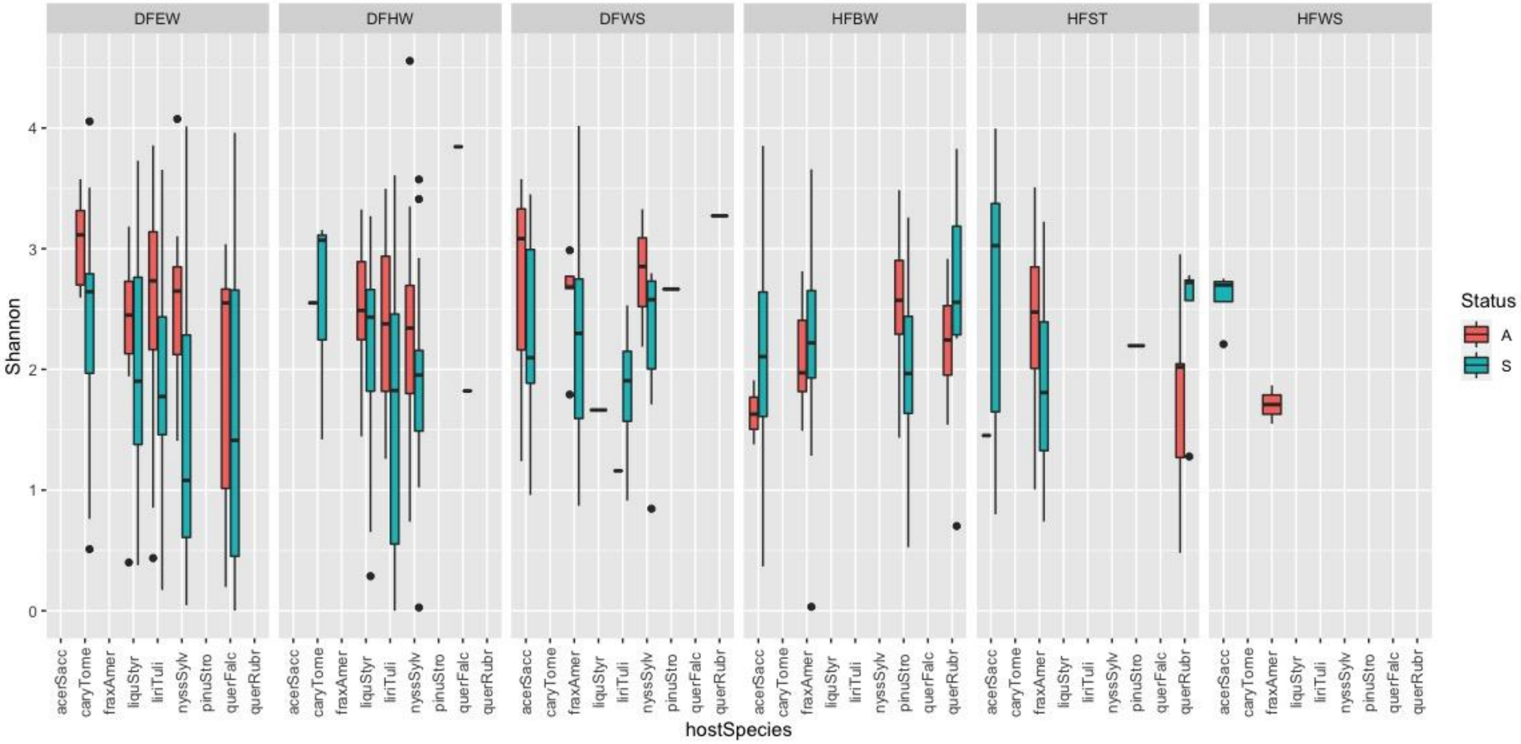
DFEW
DFHW
DFWS
HFBW
HFST
HFWS
Shannon
0
1
2
3
4
acerSacc
caryTome
fraxAmer
liquStyr
liriTuli
nyssSylv
pinuStro
querFalc
querRubr
hostSpecies
Status
A
S

Shannon

Figure S1D. Sensitivity of fungal OTUs and host health status to experimental components. Levels of these components are described in Table S3. Boxplots of sensitivity are labeled according to geographic origin of seed: seednorth/seedsouth; type of plant tissue analyzed: stem (stem only); with root (root only or root+stem); DNA extraction methodology: DNAextCTAB or DNAextkit (CTAB or DNEasy kit, see description in Methods section); sampling year (2010-2013); Sequencing methodology (MiSeq/Roche454). Data from the studied nine host was analyzed in gjam v 2.32 including OTUs found in at least 10% of the samples.

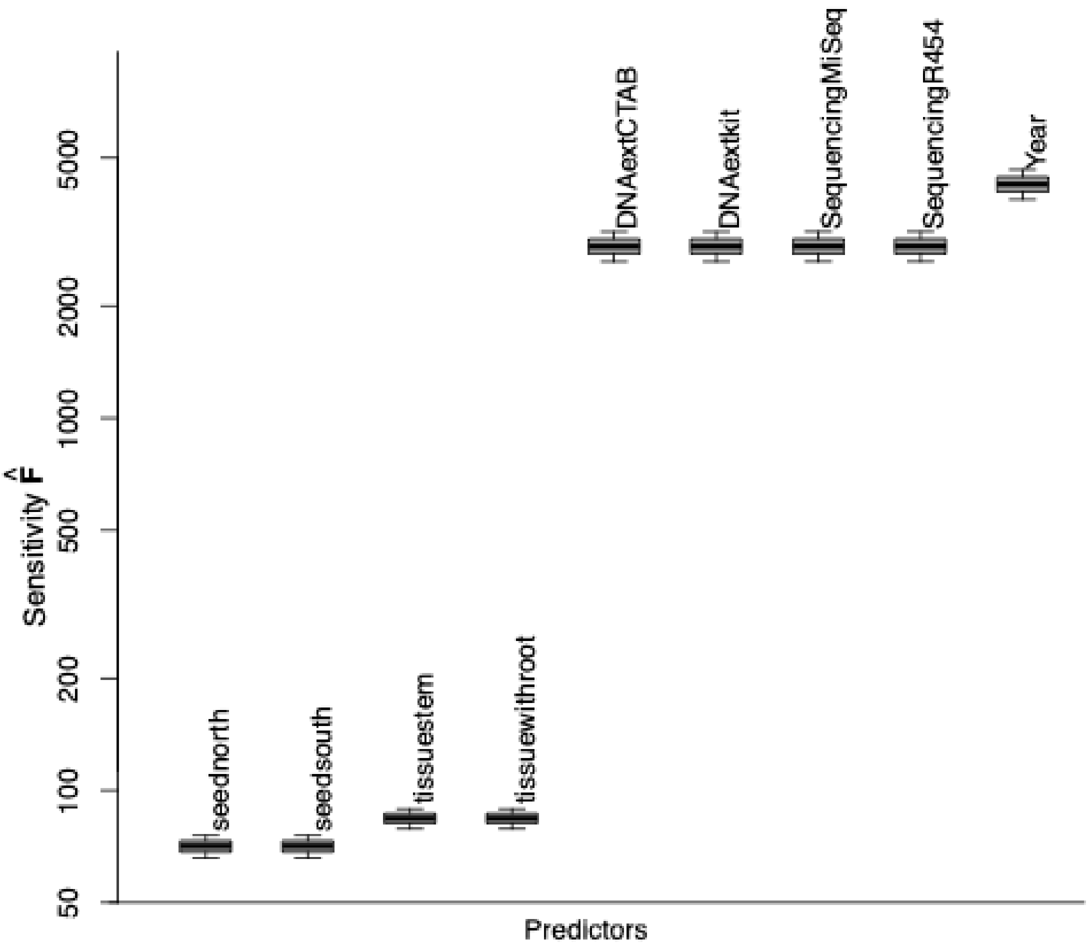

Figure S1E. Host x density responses. Posterior distributions of fungal OTUs response to host species and their interactions with density of conspecific seedlings. In all figures, bars above the dotted line represent taxa with a positive response to the low density treatment (see Table 1); bars below the line represent a negative response. Only taxa with credible intervals different than zero are shown.

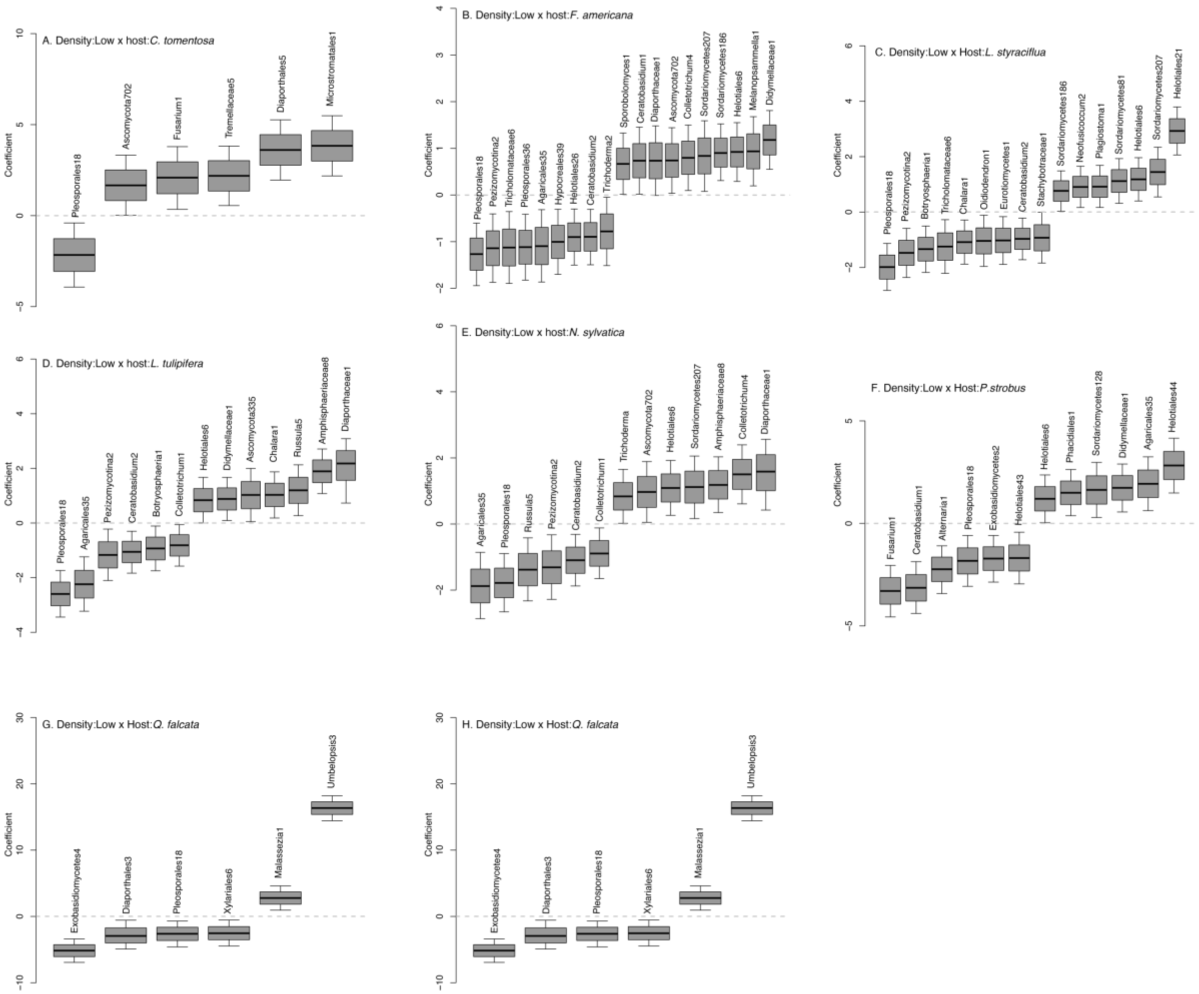

Figure S1F. Host x distance responses. Posterior distributions of fungal OTUs response to host species and their interactions with distance from conspecific adults. In all figures, bars above the dotted line represent taxa with a positive response to the near treatment (<10 m from a conspecific adult); bars below the line represent a negative response. Only taxa with credible intervals different than zero are shown.

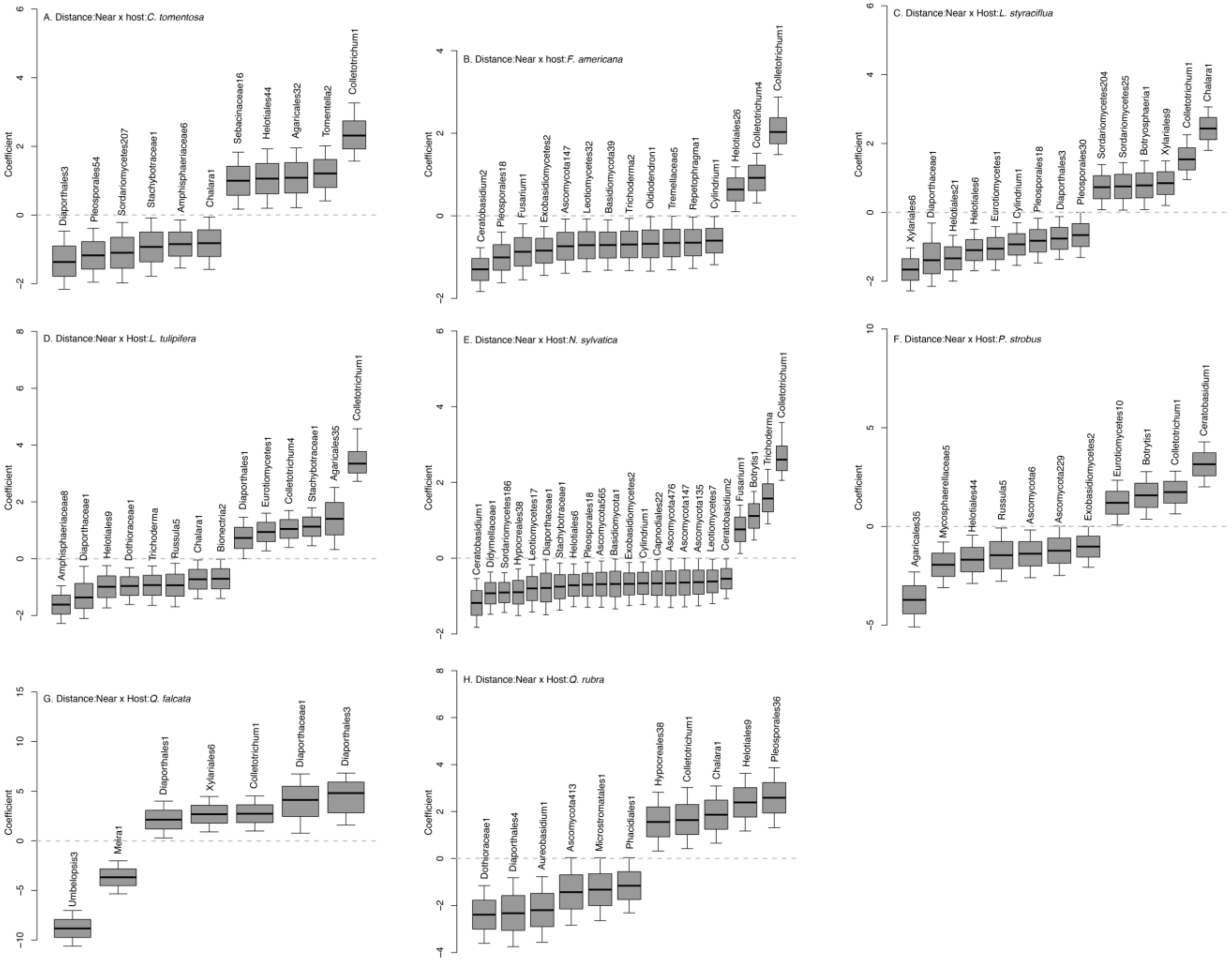

Figure S1G. Site and abiotic responses. Posterior distributions of fungal OTUs responses to site (A-F) and abiotic treatments (G-H); treatments follow Table 1. Only taxa with credible intervals different than zero are shown. DFEW, DFHW, DFWS=Duke Forest Eno West, Hardwood and Warming Site, respectively; HFBW, HFST, HFWS=Harvard Forest Barre Woods, Simes Tract and Warming Site, respectively.

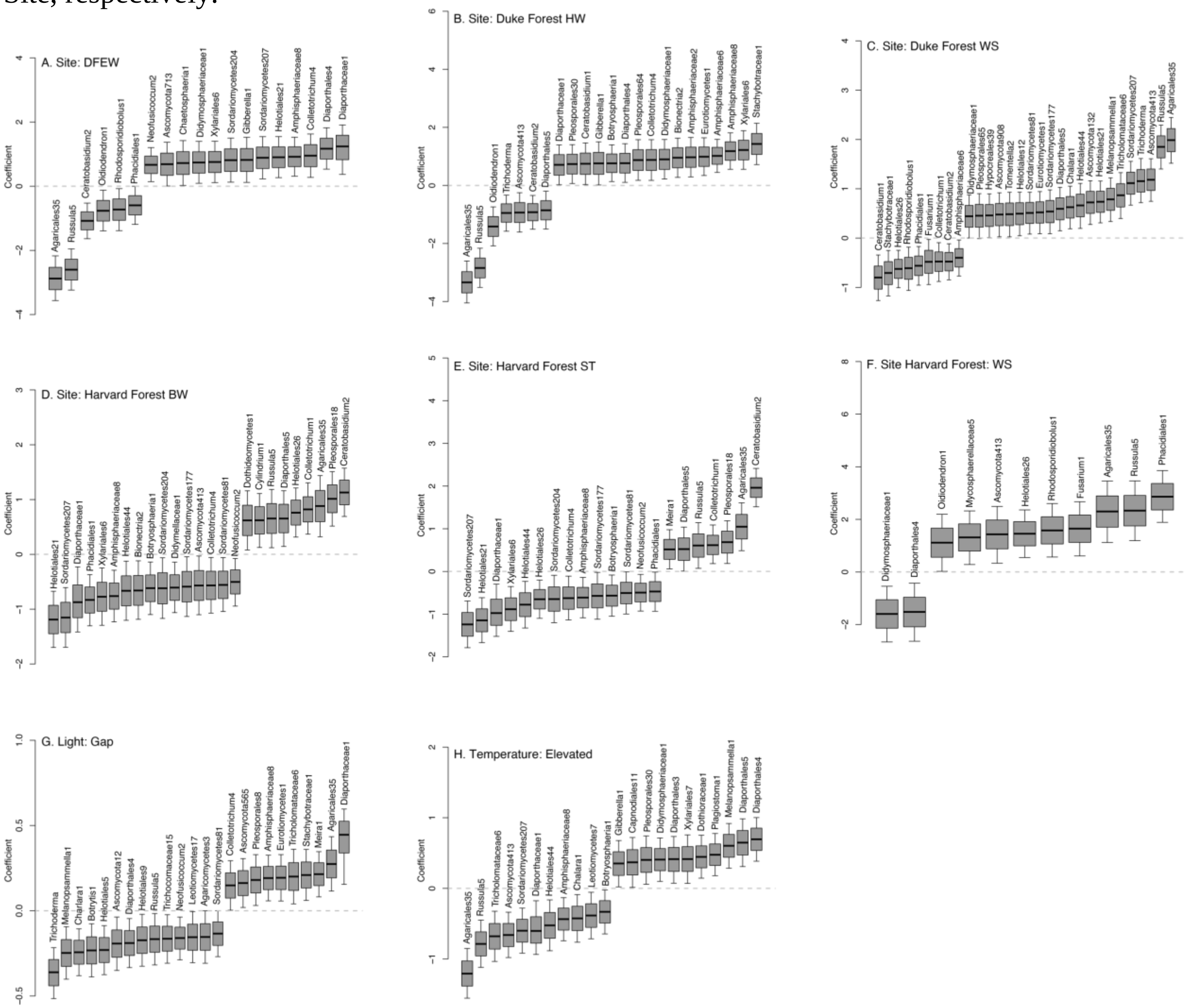

Figure S1H. Host responses. Posterior distributions of fungal OTUs responses to single hosts; see S2 table for abbreviations. Only taxa with credible intervals different than zero are shown.

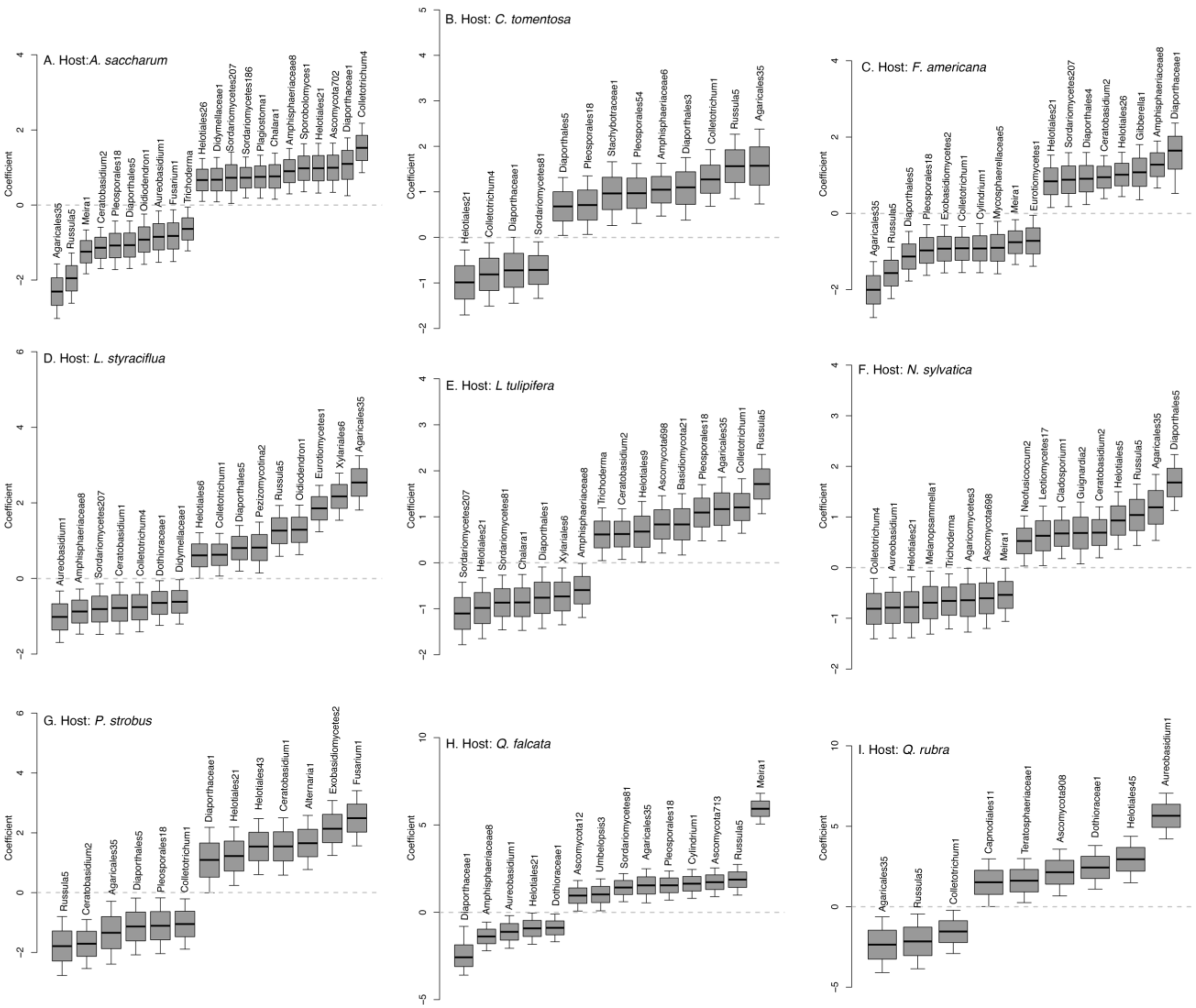

Figure S1I. Responses to year. Posterior distributions of fungal OTUs responses and host status (red) to year of study. Only taxa with credible intervals different than zero are shown.

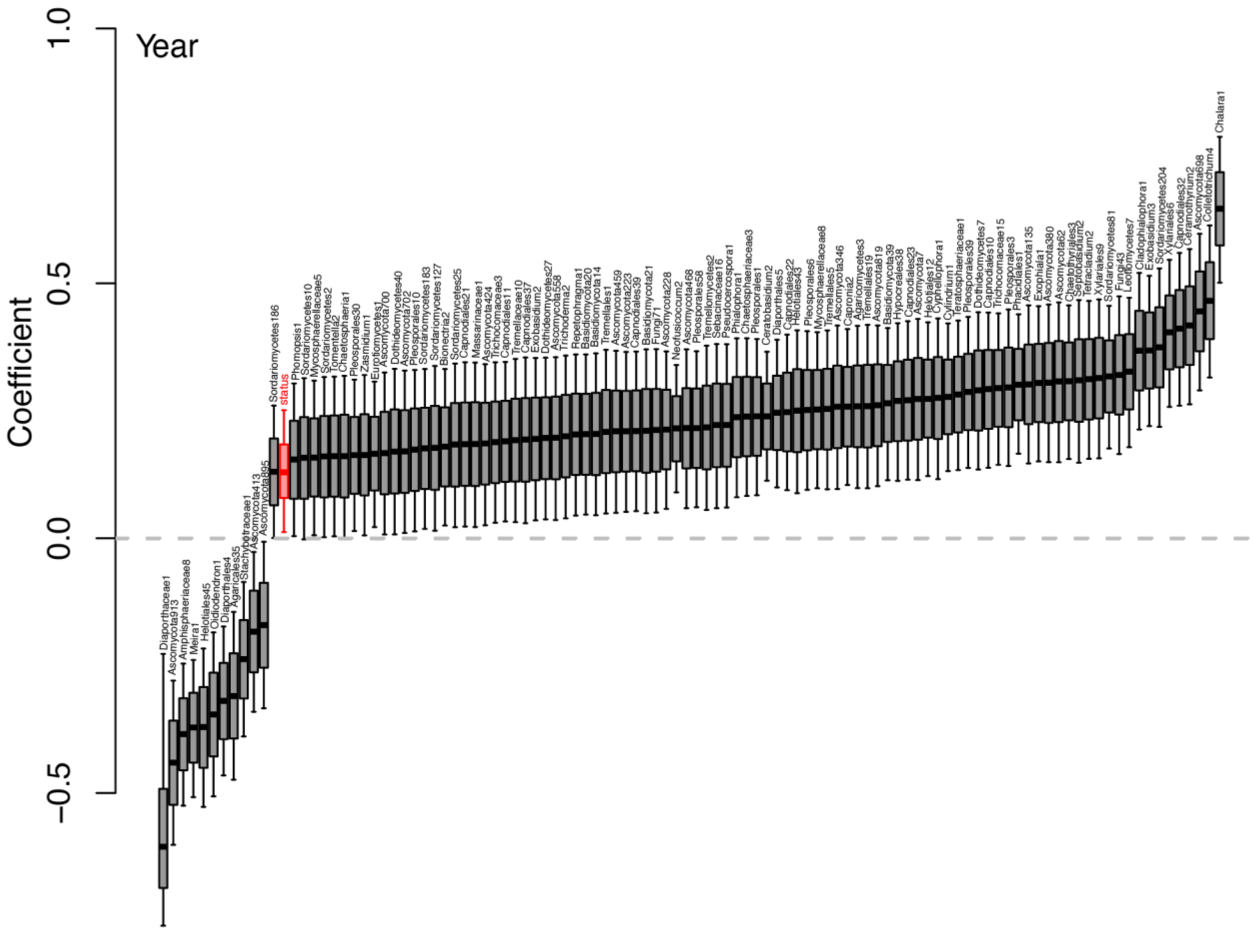

Figure S1J. Clustering of species and predictors (next page). Clustering of species responses and predictor contributions to the best model of mycobiome and host status responses to biotic (host, distance and density), abiotic (temperature, site, light) characteristics. Left panel, matrix showing clustering of response variables (fungal OTUs and host health status) according to both taxa co-occurrence and responses to predictor variables. Response variables names are shown on the left of the figure, with a hierarchical clustering image shown on top of the panel. Right panel, matrix of strength of predictor contributions to response variables. Predictors are clustered by similarity in contributions to the model (top left panel). Color scale indicates strength of responses (left panel) or contributions (right panel) to the model (blue strong negative to red strong positive), and are shown separately for each panel. Based on similarity of responses, response variables (OTUs and host status) are grouped into four clusters (marked as 1-4). A list of taxa in each cluster is found in Table S10.

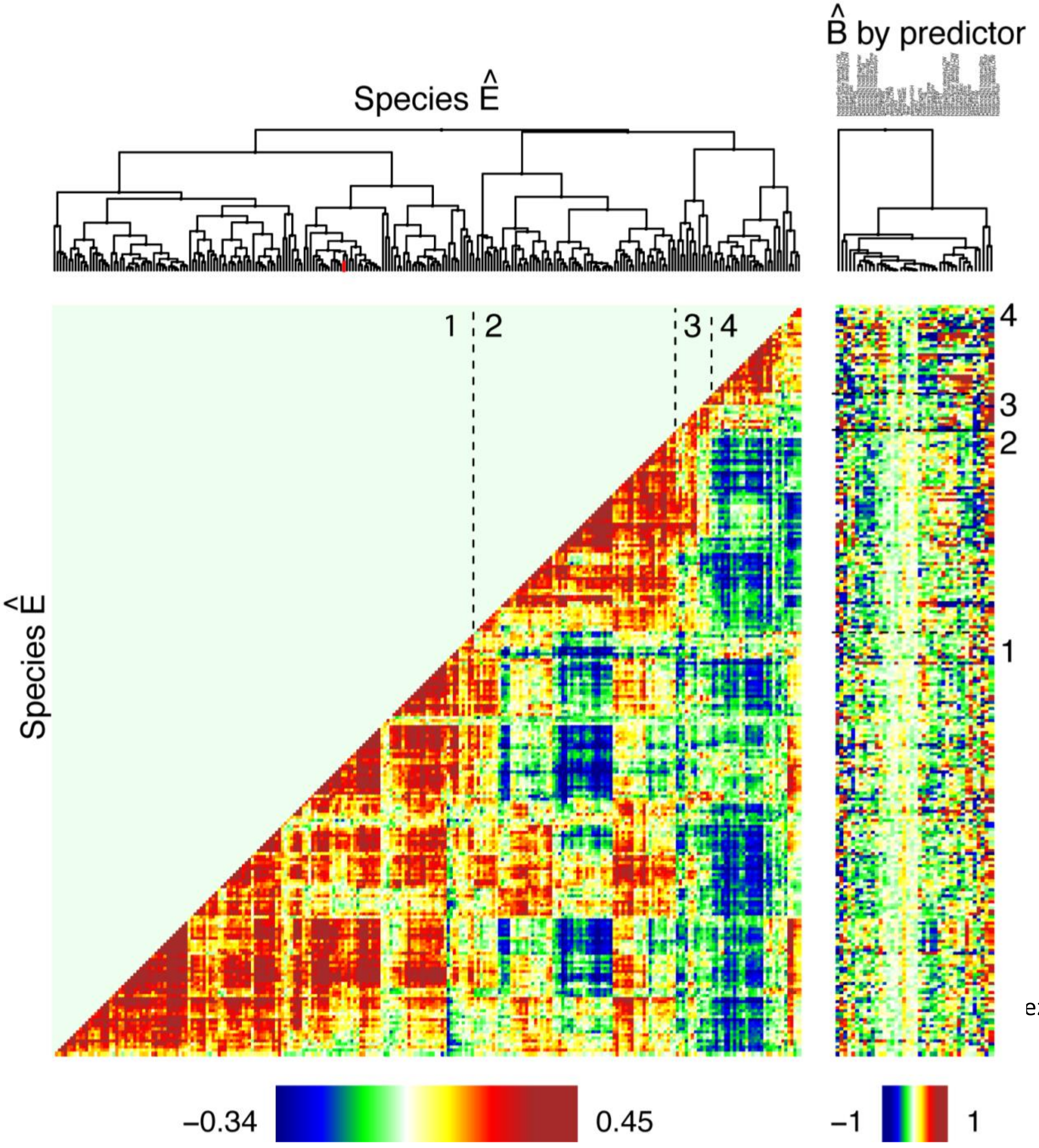

Species $\hat{E}$
$\hat{B}$ by predictor
1
2
3
4
4
3
2
1
Species $\hat{E}$
−0.34
0.45
−1
1
