# Supplementary material for: Fungal community and taxa specialization to host and environment interactions in two temperate forests
Source: PLoS One. 2025 May 9;20(5):e0322440. doi: 10.1371/journal.pone.0322440 (PMC12063886; doi:10.1371/journal.pone.0322440)
Supplement: S2 File — (PDF) [file pone.0322440.s002.pdf]

Fungal community and taxa specialization to host and environment interactions in two temperate forests

Maria Soledad Benitez Ponce, Michelle H. Hersh, Lindsey Becker, Rytas Vilgalys, James S. Clark

S2 File. Supporting Tables S2A-S2J

Corresponding author:
Maria Soledad Benitez Ponce
e-mail: benitezponce.1@osu.edu

Table S2A. Description of experimental sites

Forest	Coordinates	Mean annual temp.	Annual precipitation	Dominant forest composition	Soil characteristics	Site notes
**Duke Forest**	36.0°N, 79.1°W	14.5	1208 mm	Uneven aged Mixed hardwood and conifer (secondary growth)	Sandy loam/loam	EnoWest division is a xeric upland; dominated by oak-hickory<br>Blackwood division is mesic; dominated by poplar-sweetgum-ash and red maple
**Harvard Forest**	42.5°N, 72.2°W	7.5	1183 mm	Mixed hardwood and conifer	Glacial till	Barrewoods and Sime division are both mesic with topographic variation

Table S2B. Tree species analyzed in this study. Seeds were planted at multiple experimental sites and first-year seedlings collected for fungal community analysis. Seedling health status and fungal community composition were modeled in response to biotic and abiotic variables and their interactions.

Host species	Species abbreviation	Mycorrhizal status	Site and experiment
***A. saccharum* Marshall**	acerSacc	AM	DF: WS<br>HF: BW ST
***Carya alba* (Linnaeus)**	caryTome	EM	DF: EW, HW
***Fraxinus americana* L.**	fraxAmer	AM	DF: WS<br>HF: BW ST
***Liquidambar styraciflua* L.**	liquStyr	EM	DF: EW, HW, WS
***Liriodendron tulipifera* L.**	liriTuli	AM	DF: EW, HW, WS
***Nyssa sylvatica* Marshall**	nyssSylv	AM	DF: EW, HW, WS
***Pinus strobus* L.**	pinuStro	EM	DF: WS<br>HF: BW, ST
***Q. falcata* Michx.**	querFalc	EM	DF: EW, HW
***Q. rubra* L.**	querRubr	EM	DF: WS<br>HF: BW, ST

Table S2C. Description of methodological parameters tested as predictors of fungal community and host health responses.

Predictor	Description	Levels
Seed	Geographic origin of seed source	Seed source location (either purchased or collected). North (MA, NY, PA); South (NC, GA, LA, AR, CA)
Tissue	Tissue sampled for DNA extraction and further mycobiome analysis	Stem (stem only); with root (root only, or composite root and stem sample)
DNA extraction	Protocol used for DNA extraction of seedling tissue	CTAB or commercial kit (see Methodology for details)
Year	Sampling year	2010, 2011, 2012 and 2013
Sequencing	Sequencing platform used for mycobiome characterization	Roche 454 (R454)<br>Illumina MiSeq (MiSeq)

Table S2D. Comparison of GJAM model runs to test hypothesis of abiotic and biotic predictor contributions to fungal communities and host health status. Model runs were performed using data for 521 seedling samples corresponding to nine tree species (see Table S2B for host species list and Table S2C for predictor descriptions) and for 247 fungal taxa present in at least 10% of the samples. Interactions between predictors are indicated by "*" and include effect of host and distance and/or density. DIC scores for eight GJAM model runs are shown.

Biotic predictors	Abiotic predictors	Co-variate	DIC score
Distance*host, Density*host	Temperature, Site, Light	Year	-1614048.94235989
Host	Temperature, Site, Light	Year	-1601274.59086709
Density*host		Year	-1591243.0605892
Distance*host		Year	-1587978.30732849
Distance, density, host		Year	-1573912.18905525
Distance, density, host	Temperature, Site, Light	Year	-1561554.64896333
Distance*host, Density*host		Year	-1551734.14976087
Host		Year	-1548567.36764078

Table S2E. Summary of sequences analyzed per sequencing platform and OTU overlap between sequencing datasets, based on 99% sequence similarity, using the usearch_global algorithm from Edgar (2013). * Percentage of sequences within a sequencing dataset matching the other dataset.

Sequencing platform	No. samples	No. sequences	No. OTUs	OTU overlap*
Roche 454	107	87362	801	80%
MiSeq	414	5398196	2261	15%

Table S2F. Summary of sequencing efforts and recovered OTUs across experimental factors. Further description of factors and predictors are found in Table 1, and Supplementary Tables S2B and S2C.

			Number of sequences	Number of samples	average reads/sample	OTUs	OTUs/sample
Total			5485558	521	10528.90	2889	
Sequencing platform							
	MiSeq		5398196	414	13039.12	2261	125.97
	R454		87362	107	816.47	801	40.20
Sampling Site							
	Duke Forest						
		Eno West	1509290	159	9492.39	1879	125.79
		Hardwood	1480056	91	16264.35	1512	126.03
		Warming Site	316336	63	5021.21	1144	75.57
	Harvard Forest						
		Bare Woods	1319607	126	10473.07	1304	95.87
		Simes Tract	811332	76	10675.42	1151	99.11
		Warming Site	48937	6	8156.17	382	101.67
Year							
	2011		87362	107	816.47	801	40.20
	2012		3285749	229	14348.25	1869	115.41
	2013		2112447	185	11418.63	1921	139.05
Temperature							
	Ambient		5160950	489	10554.09	2652	108.96
	Elevated		324608	32	10144.00	971	99.09
Distance							
	Far		2630020	222	11846.94	2082	121.64
	Near		2436911	204	11945.64	2045	114.55
Light							
	Gap		1089562	131	8317.27	1846	102.11
	Undersotry		4395996	390	11271.79	2460	110.45
Host Species							
	acerSacc		1081487	112	9656.13	1402	102.05
	caryTome		406571	35	11616.31	1247	171.51
	fraxAmer		940574	100	9405.74	1405	85.55
	liquStyr		633489	67	9455.06	1349	113.99
	liriTuli		494169	61	8101.13	1358	106.39
	nyssSylv		1511340	92	16427.61	1478	118.90
	pinuStro		149105	22	6777.50	780	86.32
	querFalc		117471	11	10679.18	921	163.00
	querRubr		151352	21	7207.24	731	81.29
Host Status							
	Asymptomatic		1251407	141	8875.23	1973	119.21
	Symptomatic		4234151	380	11142.50	2471	104.33
Sampled tissue							
	Root		135588	55	2465.24	1140	58.60
	Stem		5316842	445	11947.96	2360	116.04
	Whole seedling		33128	21	1577.52	553	75.76
Single species density							
	High		3857218	311	12402.63	2217	124.94
	Low		1628340	210	7754.00	2090	83.79

Table S2G. Summary of models testing the effect of host species, health status (asymptomatic vs. symptomatic), distance and density of conspecifics on Chao1 and Shannon diversity indices for Duke and Harvard Forest samples.

Duke Forest

	**Chao1**				**Shannon (H)**			
	df	SumSq	MeanSq	p-value	df	SumSq	MeanSq	p-value
Status	1	6602	6602	0.443	1	20.598	20.598	**0.000**
Host	4	176622	44156	**0.004**	4	12.220	3.055	**0.005**
Distance	1	7278	7278	0.421	1	0.802	0.802	0.316
Density	1	149084	149084	**0.000**	1	5.568	5.568	**0.009**
Status:Host	4	19607	4902	0.781	4	1.531	0.383	0.749
Status:Distance	1	607	607	0.816	1	0.035	0.035	0.833
Host:Distance	4	86455	21614	0.106	4	7.973	1.993	**0.043**
Status:Density	1	2	2	0.989	1	0.553	0.553	0.405
Host:Density	4	71644	17911	0.175	4	1.569	0.392	0.740
Distance:Density	1	25394	25394	0.133	1	3.993	3.993	**0.026**
Status:Host:Distance	4	5866	1466	0.971	4	2.194	0.549	0.599
Status:Host:Density	2	17585	8793	0.457	2	0.711	0.355	0.640
Status:Distance:Density	1	583	583	0.820	1	0.002	0.002	0.965
Host:Distance:Density	1	23034	23034	0.153	1	1.649	1.649	0.151
Status:Host:Distance:Density	1	58	58	0.943	1	1.071	1.071	0.247
Residuals	218	2436573	11177		218	173.045	0.794	

Harvard Forest

	**Chao1**				**Shannon (H)**			
	df	SumSq	MeanSq	p-value	df	SumSq	MeanSq	p-value
Status	1	0	0	0.996	1	0.286	0.286	0.471
hostSpecies	3	60206	20069	0.056	3	5.186	1.729	**0.026**
Distance2	1	478	478	0.805	1	0.815	0.815	0.224
density	1	15	15	0.965	1	0.041	0.041	0.786
Status:hostSpecies	3	28552	9517	0.303	3	3.997	1.332	0.067
Status:Distance2	1	66	66	0.927	1	0.193	0.193	0.554
hostSpecies:Distance2	3	59241	19747	0.059	3	0.540	0.180	0.804
Status:density	1	31457	31457	**0.046**	1	0.635	0.635	0.283
hostSpecies:density	3	21593	7198	0.430	3	1.188	0.396	0.539
Distance2:density	1	1874	1874	0.624	1	0.485	0.485	0.348

Status:hostSpecies:Distance2	2	11241	5621	0.487	2	0.448	0.224	0.665
Status:Distance2:density	1	750	750	0.757	1	0.036	0.036	0.799
hostSpecies:Distance2:density	2	8976	4488	0.563	2	0.724	0.362	0.517
Residuals	152	1182219	7778		152	83.195	0.547	

Table S2H. Number of fungal taxa responses to individual predictors

a) Site

	DFEW	DFHW	DFWS	HFBW	HFST	HFWS
Positive	13	16	9	9	7	9
Negative	6	7	21	16	14	2

b) Light availability

	Open gap
Positive	10
Negative	14

c) Temperature treatment

	Elevated temperature
Positive	11
Negative	11

d) Density of conspecifics

	High
Positive	6
Negative	6

e) Distance to conspecific adult

	Near
Positive	8
Negative	4

Table S2I. Fungal OTU members of each of the four clusters sharing similarity in responses to biotic (host, distance and density to conspecifics), abiotic predictors (site, temperature, light availability) and sequencing platform (see Figure S1J).

Cluster	Fungal OTUs
1	Tomentella2, Ascomycota700, Pleosporales52, Ascomycota371, Eurotiomycetes10, Pleosporales64, Amphisphaeriaceae2, Hypocreales37, Stachybotraceae1, Ascomycota557, Colletotrichum1, Xylariales5, Hypocreales7, Polyporales2, Sordariomycetes76, Sordariomycetes128, Amphisphaeriaceae7, Ascomycota565, Sordariomycetes55, Sordariomycetes187, Xylariaceae1, Pleosporales30, Dothideomycetes23, Davidiellaceae1, Amphisphaeriaceae6, Amphisphaeriaceae3, Dothideomycetes55, Ascomycota4, Umbelopsis3, Malassezia1, Malassezia2, Ascomycota811, Zasmidium1, Ascomycota223, Ascomycota802, Mycosphaerellaceae8, Sordariomycetes14, Xylariales6, Eurotiomycetes1, Ascomycota249, Ustilaginaceae2, Capnodiales8, Ascomycota697, status, Pleosporales65, Bionectria2, Botryosphaeria1, Guignardia2, Dothideomycetes24, Curreya1, Pleosporales27, Pleosporales1, Ascomycota713, Teratosphaeriaceae1, Cladosporium1, Microstromatales1, Oomycetes12, Ascomycota819, Ascomycota698, Colletotrichum4, Basidiomycota21, Basidiomycota28, Botrytis1, Hypocreales2, Chaetosphaeriaceae3, Tainosphaeria1, Hypocreales38, Pleosporales58, Capnodiales37, Phialophora1, Ascomycota7, Cladophialophora1, Pleosporales3, Fusarium1, Pleosporales54, Capnodiales11, Sordariomycetes106, Exobasidium2, Exophiala1, Capnodiales23, Capnodiales10, Tremellales5, Phomopsis1, Sordariomycetes11, Sordariomycetes2, Trichoderma2, Dothideomycetes27, Sordariomycetes127, Mortierella4, Ascomycota228, Ascomycota82, Helotiales9, Capnodiales21, Ascomycota135, Ascomycota12, Ascomycota459, Ascomycota346, Ascomycota558, Sordariomycetes204, Fungi71, Fungi43, Septobasidium2, Neofusicoccum2, Chaetosphaeria1, Sordariomycetes25, Sordariomycetes81, Diaporthales5, Dothideomycetes40, Ascomycota380, Capnodiales39, Dothideomycetes7, Ascomycota816, Capronia2, Tetracladium2, Pleosporales6, Ascomycota62, Xylariales7, Herpotrichiellaceae19, Helotiales12, Sebacinaceae16, Rhodotorula1, Ceramothyrium2, Exobasidium3, Tremellales19, Pseudocercospora1, Agaricomycetes3, Tremellomycetes2, Tremellales1, Trichocomaceae15, Trichocomaceae3, Basidiomycota20, Umbelopsis2, Leotiomycetes17, Ascomycota469, Ascomycota172, Chalara1, Helotiales5, Ascomycota667, Helotiales18, Sordariomycetes151

2	Pleosporales18, Pleosporales17, Ascomycota686, Massarinaceae1, Ascomycota23, Phacidiales1, Oomycetes10, Sordariomycetes183, Sordariomycetes44, Sordariomycetes84, Pleosporales39, Pleosporales10, Ascomycota630, Helotiales13, Basidiomycota39, Chaetothyriales3, Tremellaceae10, Capnodiales22, Basidiomycota14, Cylindrium1, Pleosporales12, Sordariomycetes186, Ascomycota694, Rhodosporidiobolus1, Basidiomycota7, Leotiomycetes32, Ascomycota64, Exobasidiomycetes2, Ascomycota147, Ascomycota6, Ascomycota229, Ascomycota702, Mycosphaerellaceae5, Basidiomycota1, Helotiales25, Dothideomycetes1, Helotiales26, Neofabraea3, Helotiales6, Leotiomycetes2, Spizellomycetales1, Ascomycota468, Ascomycota242, Ascomycota483, Ceratobasidium1, Helotiales11, Helotiales43, Leotiomycetes7, Ascomycota424, Capnodiales32, Ascomycota476, Cyphellophora1, Repetophragma1, Agaricomycetes102, Hilberina1, Ascomycota793, Ascomycota236, Ceratobasidium2, Pleosporales36, Pleosporaceae1, Sordariomycetes10, Ascomycota780, Sordariomycetes179, Botryosphaeriaceae3, Sordariomycetes165, Diaporthales3, Sordariomycetes3
3	Aureobasidium1, Dothioraceae1, Phaeomoniella1, Didymosphaeriaceae1, Alternaria1, Dothideomycetes60, Tremellaceae5, Myringiales1, Didymellaceae1, Diaporthales1, Plagiostoma1, Hypocreales39
4	Umbelopsis4, Ascomycota335, Sordariomycetes177, Ascomycota132, Meira1, Pezizomycotina2, Trichoderma, Russula5, Agaricales32, Sordariomycetes207, Helotiales21, Helotiales44, Ascomycota913, Ascomycota413, Ascomycota908, Ascomycota895, Agaricales35, Oidiodendron1 Tricholomataceae6, Pleosporales8, Ascomycota839, Helotiales45, Ascomycota896, Diaporthaceae1, Amphisphaeriaceae8, Diaporthales4, Trichocomaceae1, Gibberella1, Melanopsammella1
