# Supplementary material for: Fungal community and taxa specialization to host and environment interactions in two temperate forests
Source: PLoS One. 2025 May 9;20(5):e0322440. doi: 10.1371/journal.pone.0322440 (PMC12063886; doi:10.1371/journal.pone.0322440)
Supplement: S3 File — (PDF) [file pone.0322440.s003.pdf]

Fungal community and taxa specialization to host and environment interactions in two temperate forests

Maria Soledad Benitez Ponce, Michelle H. Hersh, Lindsey Becker, Rytas Vilgalys, James S. Clark

S3 File. Supplementary Methods

Corresponding author:
Maria Soledad Benitez Ponce
e-mail: benitezponce.1@osu.edu

S2 Text File: Methods.

Seedling collection and fungal community characterization

To evaluate parameters impacting fungal communities and host specificity in forest seedlings, seedlings were planted and collected from experimental forest plots in the eastern USA. Two sites were studied at each Duke Forest (North Carolina, USA) and Harvard Forest (Massachusetts, USA). The Edeburn Division of Duke Forest (DF) in Orange County North Carolina (36.0°N, 79.1°W; elevation 180 m) has a mean annual temperature of 14.5 °C and annual precipitation of 1208 mm. Harvard Forest (HF) in Petersham, Massachusetts (42.5°N, 72.2°W; elevation 340 m) has a mean annual temperature of 7.5 °C and 1183 mm of precipitation; snow cover is typical in the winter months. For each forest location, two sites with variation in soil moisture were selected. At Duke Forest, Eno West (DFEW) is a xeric upland, and Blackwood (DFBW) is mesic. At Harvard Forest, Barre Woods. (HFBW) and Simes (HFST) are both mesic but contain microtopographic variation that can allow for differences in soil moisture between plots (S2A Table).

At each site, forest stands were mapped, and two sets of experimental plots were established. First, plant-soil feedback plots, hereafter “PSF plots”, were established at known distance to conspecific adults and density of conspecifics (S1A Figure); second, elevated temperature (or “warming”) plots were established within existing experimental warming chambers (as described in [1]). The PSF plots consisted of a series of 1-m^2 herbivore-enclosure cages located at known distances from conspecific adult trees. For each host species, far distance was considered as > 10 meters from a conspecific adult. Seeds were planted in each PSF plot at two levels of conspecific planting density. “High density” plots consisted of all planting positions (80 planted positions/ 1-m^2 plot) being planted with the same tree species; whereas “low density” plots were planted with multiple species per plot (five host species, 16 planting position per species, 80 planted positions/ 1-m^2 plot). The warming plots were setup within 17-m^2 rectangular open-top chambers, with air and soil warming treatments. Multiple tree species were planted each year at each warming plot, with up to 420 planting locations per chamber. In addition, for both sets of experimental plots, light was managed by plot location and setup in the understory or under opened gaps.

Seeds were planted in the PSF or the warming plots during late fall/early winter, prior to ground freezing, depending on each year's weather. First-year seedlings were monitored for seedling germination and survival and sampled for fungal communities' analysis in the growing seasons of 2011, 2012, and 2013. At each experimental site, seeds were planted during the fall/winter and monitored/sampled the following spring/summer. Seeds were locally sourced, when possible, through commercial seed suppliers or collected by team members (NC or MA). Seedling germination and survival during the growing season was monitored with weekly inventories at each site in all years. Seedlings were sampled for fungal communities' analysis if they exhibited symptoms of pathogen, pest or abiotic damage (e.g. leaf damage and discoloration, chlorosis, wilting, stem damage, canker; collectively labeled as symptomatic seedling). Each time a symptomatic seedling was collected, an asymptomatic, conspecific neighbor, if available, was also collected. Seedlings were sampled using a garden trowel, minimizing plot damage and maximizing root recovery. Collected seedlings were kept cool (4-8 °C), or shipped frozen (from HF to Duke University), until processing in the laboratory (~ 24-48 hours after sampling).

Fungal community characterization

In the laboratory, seedlings were washed in tap water and blotted on paper towels. Stem and root tissue were processed separately, and leaves were pressed and air dried. Stem and root tissue were surface sterilized through a series of washes in 70% ethanol for 60 seconds, 1% sodium hypochlorite for 60 seconds, 70% ethanol for 30 seconds, followed by a rinse in sterile distilled water. After surface sterilization, seedling tissue was quickly frozen in liquid nitrogen and stored at -80 °C. Immediately prior to DNA extraction, seedling tissue was vacuum-dried and ground to fine powder using 4 mm sterile stainless-steel beads and 1-minute bead-beating in a Genogrinder 2000 (SpexSample Prep, Metuchen, NJ). Total DNA was extracted from ~ 100 mg ground tissue using either a CTAB protocol [modified from 2–4] or with the NucleoSpin-96 Plant DNA extraction kit (Machere-Nagel / Clontech, Duren, Germany; with buffers PL2 and PL3). DNA extraction success and quality were confirmed through PCR amplification for fungal markers for the large subunit of the ribosomal rDNA, prior to amplicon library preparation.

The seedling fungal communities was surveyed through amplicon sequencing of the large subunit of the ribosomal gene with primers LROR and LR3 [4,5] in either 454-pyrosequencing

(2011 samples) or Illumina MiSeq (2012 and 2013 samples) sequencing technologies. The LROR and LR3 primer sets were selected due to consistent amplification of fungal OTUs across the multiple plant species studied. For 454-pyrosequencing library preparation, primer constructs were generated using fusion adapter A sequence + 10 base-barcode sequences + gene-specific forward primer (LROR) and were ordered through integrated DNA technologies (IDT, Coralville, IA). The reverse primer consisted of fusion adapter B sequence + gene-specific reverse primer (LR3). The PCR reaction was setup using 5 ml of CTAB-extracted DNA template in a total 25 ml reaction containing 1.5 mM MgCl2 and 0.625 uM each primer construct, 1 mg/ml BSA, 0.2 mM dNTPs and 5U Platinum Taq (Invitrogen/ThermoFisher Scientific, Waltham, MA, USA). The PCR amplification program consisted of 30 cycles with annealing temperatures of 52 °C and two-minute annealing and extension steps. PCR products were visualized in 1.5% agarose gel electrophoresis and band intensity was scored into three categories. Positive PCR products were pooled into one sample based on band intensity. Pooled PCR reactions were cleaned and concentrated using Qiagen's Minispin column PCR cleanup kit (Qiagen, Hilden, Germany). Pyrosequencing was performed at the Duke University Core Genomics Facility using FLX titanium chemistry and a Roche genome sequencer. There pooled amplicons were cleaned with AmpureBeads XP (Beckman Coulter, Brea, CA, USA) and quality checked using Agilent's Bioanalyzer instrument, prior to dilution and preparation for emulsion PCR and sequencing using facility protocols. Samples were run in multiple sets of 1/8 454-pyrosequencing reactions.

Amplicon library preparation for Illumina MiSeq was performed following modifications from [6]. The library preparation protocol consisted of three subsequent PCR reactions. The first PCR comprised of 10 cycles with regular (non-tagged) gene-specific primers (LROR-LR3, [4,5]), using either 2.5 ml of CTAB-extracted DNA or 1 ml of kit extracted template, in a 12.5 ml reaction containing 0.5 mM each primer and 1.5 mM MgCl2. The second round of PCR was performed using a mixture of six frameshift-tagged LROR-LR3 primers, each primer containing also a linker sequence for the third PCR reaction. The second round of PCR with the frame-shift primers followed the same PCR protocol as with the regular gene-specific primers and used 2.5 ml PCR product (from round 1) as template for a 12.5 ml reaction. The third PCR reaction involved universal primers that will recognize the linker region used in round 2, and also

incorporated Illumina adapter sequences and 10-base pair barcodes (added on the reverse primer only). The third PCR reaction was setup in 25 ml reactions, with 10 ml template from step 2 PCR. The amplification program consisted of 10 cycles with a 2-minute annealing step at 63 °C and 2 min extension, followed with a 10-minute final extension step at 72 °C. All PCR reactions were performed with Invitrogen's Platinum Taq polymerase. Individual PCR products were visualized and quantified using Qiagen's QIAxcel system, with the DNA screening cartridge. PCR products were then pooled in equimolar concentrations and the pooled sample was purified twice with Ampure XP beads at a 0.8:1 bead:template ratio. Amplicon libraries were sequenced in an Illumina MiSeq 2x250 platform at Duke University Core Genomics Facility. All sequence and associated metadata has been deposited in Qiita [7] under study ID 12978.

For both 454 and Illumina data, sample de-multiplexing and initial quality filtering was performed using QIIME versions 1.7 and 1.8, respectively [8]. For 454 data, downstream processing included denoising using Acacia [9] version 1.52, performed individually for each 454-run. Denoised sequences were then merged and the UPARSE algorithm implemented in USEARCH version 7 [10] was used for OTU calling at 97% similarity cutoff. Initial taxonomy assignment was based on the SILVA [11] LSU reference dataset version 111. For demultiplexed Illumina sequences, frame-shift primers were removed using cutadapt version 1.3 [12], followed by quality-based filtering and truncation, sequence de-replication, OTU-calling and generation of OTU-sample tables using the USEARCH 7 pipeline [10]. Illumina and 454-generated OTUs were merged using the USEARCH 7 global alignment algorithm (usearch_global). For this, the Illumina-OTU reference sequences generated in this study were used as the database against which the 454-OTU reference sequences were queried. OTU tables were then merged by matching OTUs from both datasets, as well as keeping unique OTUs for each sequencing platform. Taxonomy assignments of merged OTUs were revised and manually curated, using SILVA's LSU release from 3-30-2015 and revised in 2022 and manually curated using BLAST when revisions were required. Fungal functional guilds were predicted for all taxa identified to genus using FUNGuild [13]. The generated OTU table was filtered to remove any non-fungal (or oomycete) sequence. A final taxa (2889 OTUs) by sample matrix (521 samples) was generated and dataset statistics, including alpha and beta-diversity estimates were calculated using the phyloseq package version 1.24.2 [14] in R. Sequence counts were used to estimate diversity

based on the Chao1and Shannon indices (*estimate_richness* command). Effects of host species, symptomatic status, distance and density on both diversity metrics were estimated using linear mixed effect models (*lm* command) with year as a random effect. Analysis was separately run per forest, as different plant species were tested at each Duke and Harvard Forest [15]. Total number of samples analyzed are summarized in Table S2F.

Models to determine fungal communities and seedling health responses to environmental variables

Multivariate analyses and models of fungal community and host responses to predictors were performed on sequence count data using the *gjam* (version 2.3.2) package in R [16]. Generalized Joint Attribute Modeling (GJAM) jointly models multivariate data, accommodating count-composition data with massive zeros, hence it is ideal for amplicon sequence-based microbial community surveys obtained from different sequencing platforms. Because different data types are modeled on the observation scale, the coefficients and covariance/correlation matrices have direct interpretation. For this application, it allows to jointly model composition data for OTUs together with binary host responses (symptomatic and asymptomatic). A model selection approach was used to determine biotic and abiotic predictors of fungal community composition and host health status. All models included host identity as a predictor, as effective specialization has been observed for seedling fungal communities in this system [17]. Model combinations were built with variables known to affect seedling survival and fungal colonization in forest ecosystems (S2D Table), including components of distance and density dependent regulation of plant community composition; as well as methodological choices, such as DNA extraction method and sequencing platform that are known to affect the sequence data recovered from an amplicon survey approach [18]. We ran preliminary tests with models that included only methodological factors, specifically sequencing platform, sampling year, DNA extraction method, tissue type, and seed origin (S2C Table), to determine if any method may have influenced the data.

For the analysis in GJAM, seedling samples from the 9 host species planted in the PSF cages were analyzed (S2B Table). OTUs were filtered to included only those found in a minimum of 10% of the samples analyzed, resulting in a total of 247 fungal OTUs (plus status and other) recovered from 521 samples were included in all GJAM analyses. Analysis with 9

hosts considered individual effects of predictors (Table 1), including host species, abiotic parameters (light availability: gap vs. understory; temperature treatment: ambient vs. elevated; site: two sites at each Duke Forest and Harvard Forest), biotic parameters (distance to adult tree and density of conspecifics), and sequencing platform (S2C Table). All models were run with *Acer saccharum* as the reference factor level for the host variable and "low density" as reference for conspecific density. Models were evaluated based on their associated deviance information criterion (DIC) scores, and model with the lowest DIC score was chosen as the best model.

References

1. Clark JS, Salk C, Melillo J, Mohan J. Tree phenology responses to winter chilling, spring warming, at north and south range limits. Functional ecology. 2014;28: 1344–1355.

2. Gardes M, Bruns TD. ITS primers with enhanced specificity for basidiomycetes - application to the identification of mycorrhizae and rusts. Molecular Ecology. 1993;2: 113–118.

3. McNickle GG, Cahill JF, Deyholos MK. A PCR-based method for the identification of the roots of 10 co-occurring grassland species in mesocosm experiments. Botany. 2008;86: 485–490. doi:10.1139/B08-014

4. Johansen RB, Johnston P, Mieczkowski P, Perry GL, Robeson MS, Burns BR, et al. A native and an invasive dune grass share similar, patchily distributed, root-associated fungal communities. Fungal Ecology. 2016;23: 141–155.

5. Bonito G, Reynolds H, Robeson MS, Nelson J, Hodkinson BP, Tuskan G, et al. Plant host and soil origin influence fungal and bacterial assemblages in the roots of woody plants. MolEcol. 2014;23: 3356–3370. doi:10.1111/mec.12821

6. Lundberg DS, Lebeis SL, Paredes SH, Yourstone S, Gehring J, Malfatti S, et al. Defining the core Arabidopsis thaliana root microbiome. Nature. 2012;488: 86.

7. Gonzalez A, Navas-Molina JA, Kosciolek T, McDonald D, Vázquez-Baeza Y, Ackermann G, et al. Qiita: rapid, web-enabled microbiome meta-analysis. Nature Methods. 2018;15: 796–798. doi:10.1038/s41592-018-0141-9

8. Caporaso JG, Kuczynski J, Stombaugh J, Bittinger K, Bushman FD, Costello EK, et al. QIIME allows analysis of high-throughput community sequencing data. Nat Methods. 2010;7: 335–336. doi:10.1038/nmeth.f.303

9. Bragg L, Stone G, Imelfort M, Hugenholtz P, Tyson GW. Fast, accurate error-correction of amplicon pyrosequences using Acacia. Nat Meth. 2012;9: 425–426.

10. Edgar RC. UPARSE: highly accurate OTU sequences from microbial amplicon reads. Nat Meth. 2013;10: 996–998.

11. Quast C, Pruesse E, Yilmaz P, Gerken J, Schweer T, Yarza P, et al. The SILVA ribosomal RNA gene database project: improved data processing and web-based tools. Nucleic Acids Res. 2013;41: D590-6. doi:10.1093/nar/gks1219

12. Martin M. Cutadapt removes adapter sequences from high-throughput sequencing reads. EMBnet.journal. 2011;17: 10. doi:10.14806/ej.17.1.200

13. Nguyen NH, Song Z, Bates ST, Branco S, Tedersoo L, Menke J, et al. FUNGuild: An open annotation tool for parsing fungal community datasets by ecological guild. Fungal Ecology. 2016;20: 241–248. doi:10.1016/j.funeco.2015.06.006

14. McMurdie PJ, Holmes S. phyloseq: An R Package for Reproducible Interactive Analysis and Graphics of Microbiome Census Data. Watson M, editor. PLoS ONE. 2013;8: e61217. doi:10.1371/journal.pone.0061217

15. Oksanen J, Blanchet F, Friendly M, Kindt R, Legendre P, Mcglinn D, et al. vegan: Community Ecology Package. R package. 2016;version 2.4-1. https://CRAN.R-project.org/package=vegan.

16. Clark JS, Nemergut D, Seyednasrollah B, Turner PJ, Zhang S. Generalized joint attribute modeling for biodiversity analysis: median-zero, multivariate, multifarious data. Ecological Monographs. 2017;87: 34–56. doi:10.1002/ecm.1241

17. Hersh MH, Vilgalys R, Clark JS. Evaluating the impacts of multiple generalist fungal pathogens on temperate tree seedling survival. Ecology. 2012;93: 511–520.

18. Song Z, Schlatter D, Gohl DM, Kinkel LL. Run-to-Run Sequencing Variation Can Introduce Taxon-Specific Bias in the Evaluation of Fungal Microbiomes. Phytobiomes Journal. 2018;2: 165–170. doi:10.1094/PBIOMES-09-17-0041-R
