# Supplementary material for: Fungal community and taxa specialization to host and environment interactions in two temperate forests
Source: PLoS One. 2025 May 9;20(5):e0322440. doi: 10.1371/journal.pone.0322440 (PMC12063886; doi:10.1371/journal.pone.0322440)
Supplement: Attachment — Submitted filename: PONE-D-24-34035 reviewer.pdf [file pone.0322440.s005.pdf]

# PLOS ONE

# Fungal community and taxa specialization to host and environment interactions in two temperate forests

--Manuscript Draft--

| | |
|---|---|
| **Manuscript Number:** | PONE-D-24-34035 |
| **Article Type:** | Research Article |
| **Full Title:** | Fungal community and taxa specialization to host and environment interactions in two temperate forests |
| **Short Title:** | Fungal specialization to host and environment in two temperate forests |
| **Corresponding Author:** | Maria Soledad Benitez Ponce<br>The Ohio State University<br>Wooster, UNITED STATES OF AMERICA |
| **Keywords:** | mycobiome, host-specialization, density and distance dependence, warming |
| **Abstract:** | The structure and function of plant-associated fungal communities (i.e. mycobiome) is shaped by biotic and abiotic factors, and can impact plant community dynamics. We evaluated the effects of different environmental factors in structuring the communities of seedling-associated fungi in temperate tree species, considering both the Janzen-Connell hypothesis as well as the impacts of climate warming. We tested the hypothesis that fungal host-specialization is observed at both the individual fungus and fungal community levels and is modulated by environmental conditions. The seedling fungal communities were characterized from tree species grown in two forests, under experimental manipulation of light, warming, and distance to and density of conspecifics. Fungal communities were analyzed using generalized joint attribute models. While warming, light, and forest site played a role in structuring seedling fungal communities, host, distance to, and density of conspecifics were stronger contributors. Furthermore, we could identify which fungal taxa responded to which predictors. This work supports the concept of fungal host-specialization at the community level, and points to particular fungal taxa which may play roles in density- and distance-dependent regulation of plant species diversity in the studied forests. |
| **Order of Authors:** | Maria Soledad Benitez Ponce |
| | Michelle H. Hersh |
| | Lindsey Becker |
| | Rytas Vilgalys |
| | James S. Clark |
| **Additional Information:** | |
| **Question** | **Response** |
| **Financial Disclosure**<br><br>Enter a financial disclosure statement that describes the sources of funding for the work included in this submission. Review the submission guidelines for detailed requirements. View published research articles from *PLOS ONE* for specific examples.<br><br>This statement is required for submission and **will appear in the published article** if the submission is accepted. Please make | Yes |

*Powered by Editorial Manager® and ProduXion Manager® from Aries Systems Corporation*

| | |
|---|---|
| sure it is accurate.<br><br>**Funded studies**<br>Enter a statement with the following details:<br>• Initials of the authors who received each award<br>• Grant numbers awarded to each author<br>• The full name of each funder<br>• URL of each funder website<br>• Did the sponsors or funders play any role in the study design, data collection and analysis, decision to publish, or preparation of the manuscript?<br><br>Did you receive funding for this work? | |
| Please add funding details.<br>as follow-up to "**Financial Disclosure**<br><br>Enter a financial disclosure statement that describes the sources of funding for the work included in this submission. Review the submission guidelines for detailed requirements. View published research articles from *PLOS ONE* for specific examples.<br><br>This statement is required for submission and **will appear in the published article** if the submission is accepted. Please make sure it is accurate.<br><br>**Funded studies**<br>Enter a statement with the following details:<br>• Initials of the authors who received each award<br>• Grant numbers awarded to each author<br>• The full name of each funder<br>• URL of each funder website<br>• Did the sponsors or funders play any role in the study design, data collection and analysis, decision to publish, or preparation of the manuscript?<br><br>Did you receive funding for this work?" | National Science Foundation grant NSF-DEB-0955904 to RV and JC |
| Please select the country of your main research funder (please select carefully | UNITED STATES - US |

| | |
|---|---|
| as in some cases this is used in fee calculation).<br>as follow-up to "**Financial Disclosure**<br><br>Enter a financial disclosure statement that describes the sources of funding for the work included in this submission. Review the submission guidelines for detailed requirements. View published research articles from *PLOS ONE* for specific examples.<br><br>This statement is required for submission and **will appear in the published article** if the submission is accepted. Please make sure it is accurate.<br><br>**Funded studies**<br>Enter a statement with the following details:<br>• Initials of the authors who received each award<br>• Grant numbers awarded to each author<br>• The full name of each funder<br>• URL of each funder website<br>• Did the sponsors or funders play any role in the study design, data collection and analysis, decision to publish, or preparation of the manuscript?<br><br>Did you receive funding for this work?" | |
| **Competing Interests**<br><br>Use the instructions below to enter a competing interest statement for this submission. On behalf of all authors, disclose any competing interests that could be perceived to bias this work—acknowledging all financial support and any other relevant financial or non-financial competing interests.<br><br>This statement is required for submission and **will appear in the published article** if the submission is accepted. Please make sure it is accurate and that any funding sources listed in your Funding Information later in the submission form are also | The authors have declared that no competing interests exist. |

| | |
|---|---|
| declared in your Financial Disclosure statement.<br><br>View published research articles from *PLOS ONE* for specific examples.<br><br>**NO authors have competing interests**<br><br>Enter: *The authors have declared that no competing interests exist.*<br><br>**Authors with competing interests**<br><br>Enter competing interest details beginning with this statement:<br><br>*I have read the journal's policy and the authors of this manuscript have the following competing interests: [insert competing interests here]*<br><br>* typeset | |
| **Ethics Statement**<br><br>Enter an ethics statement for this submission. This statement is required if the study involved:<br><br>• Human participants<br>• Human specimens or tissue<br>• Vertebrate animals or cephalopods<br>• Vertebrate embryos or tissues<br>• Field research<br><br>Write "N/A" if the submission does not require an ethics statement.<br><br>General guidance is provided below. Consult the submission guidelines for detailed instructions. **Make sure that all information entered here is included in the Methods section of the manuscript.** | N/A |

| | |
|---|---|
| **Format for specific study types**<br><br>**Human Subject Research (involving human participants and/or tissue)**<br>• Give the name of the institutional review board or ethics committee that approved the study<br>• Include the approval number and/or a statement indicating approval of this research<br>• Indicate the form of consent obtained (written/oral) or the reason that consent was not obtained (e.g. the data were analyzed anonymously)<br><br>**Animal Research (involving vertebrate animals, embryos or tissues)**<br>• Provide the name of the Institutional Animal Care and Use Committee (IACUC) or other relevant ethics board that reviewed the study protocol, and indicate whether they approved this research or granted a formal waiver of ethical approval<br>• Include an approval number if one was obtained<br>• If the study involved *non-human primates*, add *additional details* about animal welfare and steps taken to ameliorate suffering<br>• If anesthesia, euthanasia, or any kind of animal sacrifice is part of the study, include briefly which substances and/or methods were applied<br><br>**Field Research**<br><br>Include the following details if this study involves the collection of plant, animal, or other materials from a natural setting:<br>• Field permit number<br>• Name of the institution or relevant body that granted permission | |
| **Data Availability**<br><br>Authors are required to make all data underlying the findings described fully available, without restriction, and from the time of publication. PLOS allows rare exceptions to address legal and ethical concerns. See the PLOS Data Policy and FAQ for detailed information. | Yes - all data are fully available without restriction |

*Powered by Editorial Manager® and ProduXion Manager® from Aries Systems Corporation*

| | |
|---|---|
| A Data Availability Statement describing where the data can be found is required at submission. Your answers to this question constitute the Data Availability Statement and **will be published in the article**, if accepted.<br><br>**Important:** Stating 'data available on request from the author' is not sufficient. If your data are only available upon request, select 'No' for the first question and explain your exceptional situation in the text box.<br><br>Do the authors confirm that all data underlying the findings described in their manuscript are fully available without restriction? | |
| **Describe where the data may be found in full sentences. If you are copying our sample text, replace any instances of XXX with the appropriate details.**<br><br>• If the data are **held or will be held in a public repository**, include URLs, accession numbers or DOIs. If this information will only be available after acceptance, indicate this by ticking the box below. For example: *All XXX files are available from the XXX database (accession number(s) XXX, XXX.)*.<br>• If the data are all contained **within the manuscript and/or Supporting Information files**, enter the following: *All relevant data are within the manuscript and its Supporting Information files.*<br>• If neither of these applies but you are able to provide **details of access elsewhere**, with or without limitations, please do so. For example:<br><br>*Data cannot be shared publicly because of [XXX]. Data are available from the XXX Institutional Data Access / Ethics Committee (contact via XXX) for researchers who meet the criteria for access to confidential data.*<br><br>*The data underlying the results presented in the study are available from (include the name of the third party* | All relevant data are within the manuscript and its Supporting Information files. |

| | |
|---|---|
| *and contact information or URL).*<br>• This text is appropriate if the data are owned by a third party and authors do not have permission to share the data.<br><br>* typeset | |
| Additional data availability information: | Tick here if the URLs/accession numbers/DOIs will be available only after acceptance of the manuscript for publication so that we can ensure their inclusion before publication. |

Manuscript file

Click here to access/download;Manuscript;Benitez_etal_MainText_PlosOne.d

# Fungal community and taxa specialization to host and environment interactions in two temperate forests

Maria Soledad Benitez Ponce[1], Michelle H. Hersh[2], Lindsey Becker[3,4], Rytas Vilgalys[3], James S. Clark[3,5]

[1]Department of Plant Pathology, The Ohio State University, Wooster, OH, 44691 USA

[2]Department of Biology, Sarah Lawrence College, Bronxville, NY, 10708 USA

[3]Department of Biology, Duke University, Durham, NC 27708, USA

[4]Department of Entomology and Plant Pathology, North Carolina State University, Raleigh, NC 27695, USA

[5]Nicholas School of the Environment, Duke University, Durham, North Carolina, 27708 USA

Corresponding author:

Maria Soledad Benitez Ponce

e-mail: benitezponce.1@osu.edu

phone: +1 330-202-3565

Short title: **Fungal specialization to host and environment in two temperate forests**

## Abstract

The structure and function of plant-associated fungal communities (i.e. mycobiome ) is shaped by biotic and abiotic factors, and can impact plant community dynamics. We evaluated the effects of different environmental factors in structuring the communities of seedling-associated fungi in temperate tree species, considering both the Janzen-Connell hypothesis as well as the impacts of climate warming. We tested the hypothesis that fungal host-specialization is observed at both the individual fungus and fungal community levels and is modulated by environmental conditions. The seedling fungal communities were characterized from tree species grown in two forests, under experimental manipulation of light, warming, and distance to and density of conspecifics. Fungal communities were analyzed using generalized joint attribute models. While warming, light, and forest site played a role in structuring seedling fungal communities, host, distance to, and density of conspecifics were stronger contributors. Furthermore, we could identify which fungal taxa responded to which predictors. This work supports the concept of fungal host-specialization at the community level, and points to particular fungal taxa which may play roles in density- and distance-dependent regulation of plant species diversity in the studied forests.

## Introduction

That pathogens play a key role in regulating plant diversity via conspecific negative density dependence (CNDD) is now well established (1,2). Determining which pathogens are involved in this mechanism has become more of a challenge than initially anticipated. Early studies documenting distance- and density-dependent seedling survival assumed

that these patterns were driven by host-specific natural enemies (3,4). But, as understanding of seedling fungal communities has deepened, this idea of strict host specificity has been recognized as an oversimplification, with many root-associated fungi colonizing multiple hosts and demonstrating different impacts on seedling survival based on host identity (5) or environmental context (6). Further, seedling root mycobiomes are now recognized as quite complex, with many co-occurring fungal species (7), such that assessing the role of each individual fungal species is challenging. Finally, all of these interactions take place in the context of a warming climate; warming can increase the relative abundance of soil pathogens (8,9) and the strength of CNDD (9). Here, we demonstrate an analytical approach that can identify specific fungal taxa responding to different predictors, including conspecific host density and distance as well as experimental warming, that may be critical in understanding distance and density-dependent responses in light of changing environmental context.

Patterns of plant abundance and demographic rates consistent with regulation by natural enemies (e.g. 10,11) have typically been discussed in the framework of the Janzen-Connell (JC) hypothesis (3,4). Under this framework, plant diversity is promoted by the host-specific attack from plant pests and pathogens. Seedling recruitment is limited by host-specific enemies in areas where the host is locally abundant, thereby increasing diversity. Demographic evidence shows that high host density and/or short distance from conspecific adults decreases seedling survival and recruitment rates (11–13). Diversity is maintained because ostensibly host-specific pathogens attack one species and not others,

and are thereby able to act as a biotic filter that limits recruitment of individuals of the same host.

However, a growing body of evidence does not find strict host specificity in individual fungal pathogens, but instead effective specialization (14). The requirement for host-specific pathogens (15) conflicts with observations that many of the individual fungal taxa associated with seedlings roots and stems are typically found in multiple hosts (6,16–19), but see also (20) though overall fungal community composition may differ between host species (21,22). Negative interactions between pathogens and their tree hosts could be modulated by additional interactions with beneficial mycorrhizal fungi, especially ectomycorrhizae (23–25). Further, environmental characteristics such as light (26,27) and water availability (17,28,29) could alter the nature of plant-pathogen interactions by altering fungal community structure, function, or both. These same environmental variables that alter fungal communities also directly affect plant health and plant community composition. For instance, seedling survival is differentially affected by abiotic conditions, such as light availability (30,31) and soil moisture (6), potentially contributing to niche differentiation in plants (30). Under the warmer temperatures predicted under climate change, forests may experience a greater relative abundance of soil pathogens (8,9), higher foliar disease severity (32,33) or intensification of negative feedbacks (9,32). However, other studies have found weaker feedbacks (34,35) and decreased pathogen abundance (34) under warmer temperatures. As climate warming progresses, an understanding of plant-soil feedbacks under warming becomes more

critical, yet studies exploring these feedbacks under experimental warming treatments are sparse (36).

Benítez et al. (14) hypothesized that JC effects could result from effective specialization, where pathogen specialization to a host results from the interaction of the host and pathogen with particular local conditions. If considering multiple fungal species infecting one host, effective specialization will occur as a result of the combined effect of different pathogen infections in a host. These pathogen effects need not to be additive, and may each in turn be modulated by the environmental conditions (see also 6). Therefore, different combinations of fungal infections will affect host species in different ways in a context-dependent manner.

Resolving the apparent contradictions between demographic studies that support JC and the lack of observed pathogen specificity, which does not support JC, requires analysis of plant-associated fungal communities and their joint relationships with host and environment. In this study, we test the hypothesis that fungal host specialization to tree species is observed for both individual fungal taxa and for communities of fungi, and that host specialization is modulated by environmental conditions. Seedling-associated fungal communities were intensively surveyed from experimental sites at two temperate forests, with an experimental design that incorporates treatments of light availability and elevated temperature as well as distance to and density of conspecifics. The seedling mycobiome fungal communities were characterized using high-throughput sequencing approaches. Generalized joint attribute modeling (GJAM; 37) was used to jointly predict both host

health status and the host-associated mycobiome based on common predictors. Specifically, we tested a set of *a priori* hypotheses focused on biotic and abiotic factors which could influence seedling health status and the mycobiome of a given seedling and evaluated them using a model selection approach. We centered on distance from conspecific adults and conspecific seedling density as predictors relevant to the JC hypothesis, as well as environmental factors (light, site) which could modulate JC interactions. A subset of seedlings were exposed to an experimental warming treatment that allowed for prediction of changing fungal responses to elevated temperature.

## Materials and Methods

### *Field experiment design and seedling collection*

To evaluate parameters impacting fungal communities and host specificity in forest seedlings, seedlings were planted and collected from experimental forest plots in the eastern USA. Two sites were studied at Duke Forest (North Carolina, USA) and Harvard Forest (Massachusetts, USA). Within each forest, sites differed in soil characteristics, topographic variation and dominant tree species. Additional detail for each site is provided in S1 Table, S1 Figure, and S1 Methods. At each site, two experimental plots were established. First, plots to study plant soil feedbacks (hereafter "PSF plots"), which consisted of 1-$m^2$ herbivore-exclusion cages located at known distances from conspecific adult trees, with far distance being > 10 meters. In addition, two levels of plant diversity were considered in PSF plots, monoculture and high diversity (five species per plot), with a total of 80 seedlings planted per PSF. Second, seeds of different tree species were

planted in previously established experimental warming chambers, as described in (38). The nine tree species included in this study are listed in S2 Table. Both for the PSF plots and the warming chambers replicate treatments were installed in locations with varying light, corresponding to understory or opened gaps (see example in S1 Fig). Seeds were planted in each plot/site during late fall/early winter, prior to ground freezing, depending on each year's weather. First-year seedlings were monitored for seedling germination and survival on a weekly basis during the growing season (May-August) and sampled for fungal community characterization in 2011, 2012, and 2013 at all sites. Seedlings were sampled if they exhibited symptoms of pathogen, pest or abiotic damage (e.g. leaf damage and discoloration, chlorosis, wilting, stem damage/canker; collectively labeled as symptomatic seedling). Each time a symptomatic seedling was collected, an asymptomatic, conspecific neighbor, was also collected (if available within the plot). Collected seedlings were kept cool (4-8 °C), transported to the laboratory, or shipped frozen (from HF to Duke), until processing. Seedlings were processed or frozen within 24-48 hours after sampling.

***Fungal community characterization***

In the laboratory, seedlings were washed in tap water and blotted on autoclaved paper towels. Stem and root tissue were processed separately and surface disinfested through a series of washes in 70% ethanol for 60 seconds, 1% sodium hypochlorite for 60 seconds, 70% ethanol for 30 seconds, followed by a sterile distilled water rinse. The tissue was then quickly frozen in liquid nitrogen and stored at -80 °C. Immediately prior to DNA extraction, seedling tissue was vacuum-dried and ground to fine powder using 4 mm

sterile stainless-steel beads and 1-minute bead-beating in a Genogrinder 2000 (SpexSample Prep, Metuchen, NJ). Total DNA was extracted from ~ 100 mg ground tissue using either a CTAB protocol (modified from 39–41) or with the NucleoSpin-96 Plant DNA extraction kit (Macherey-Nagel / Clontech, Duren, Germany; with buffers PL2 and PL3). DNA extraction success and quality were confirmed through PCR amplification and gel electrophoresis, prior to amplicon library preparation (41,42). The seedling mycobiota was surveyed through amplicon sequencing of the large subunit of the ribosomal rDNA with primers LROR and LR3 (41,42) in either 454-pyrosequencing (2011 samples) or Illumina MiSeq (2012 and 2013 samples) sequencing technologies. Greater detail on amplicon library preparation is described in Methods S1. All sequences and associated metadata have been deposited in Qiita (43) under study ID 12978.

For both 454 and Illumina data, sample de-multiplexing and initial quality filtering were performed using QIIME versions 1.7 and 1.8, respectively (44). For 454 data, processing of demultiplexed files included denoising using Acacia (45) version 1.52, performed individually for each sequencing run. Denoised sequences were then merged and the UPARSE algorithm (USEARCH version 7) (46) was used for OTU calling at 97% similarity. For demultiplexed Illumina sequences, frame-shift primers were removed using cutadapt version 1.3 (47), followed by quality-based filtering and truncation, sequence de-replication, OTU-calling and generation of OTU-sample tables using USEARCH 7 (46) as described above. Illumina and 454- OTUs were compared with USEARCH 7 global alignment tool (usearch_global). Illumina OTUs were used as the database against which the 454-OTUs were queried to at a 99% similarity. Illumina and 454-OTU tables were then merged to include both OTU matches between platforms

and OTUs unique to each. Taxonomy assignments of the final OTUs used in this research was based on comparison with SILVA's LSU release from 3-30-2015, revised in 2022 and manually curated using BLAST when revisions were required. Any non-fungal/oomycete sequences were removed prior to downstream analysis. The final mycobiota dataset consisted of 2889 taxa (OTUs) recovered from 521samples. Data set statistics, including alpha and beta-diversity metrics were estimated in phyloseq package version 1.24.2 (48) in R (49) (S1 Methods). Effects of host species, symptomatic status, distance and density on diversity metrics were estimated using linear mixed effect models with year as a random effect. Analysis was separately run per forest, as different plant species were tested at each Duke and Harvard Forest.

***Models to determine fungal communities and seedling health responses to environmental variables***

Fungal community and host responses to environmental variables were jointly modeled using Generalized Joint Attribute Modeling (37). Generalized Joint Attribute Modeling (GJAM) is a multivariate Bayesian analysis which allows one to jointly model different data types, in this case host health status (discrete and binary) and fungal OTUs (compositional data) measured with different sampling efforts (differences in sequencing depth per sequencing platform), while accommodating for the overrepresentation of zeros, which is common in microbial community experiments (50,51). A model selection approach was used to determine biotic and abiotic predictors of fungal community composition and host health status. Model combinations were built with variables known to affect seedling survival and fungal colonization in forest ecosystems, including

components of distance and density dependent regulation of plant community composition (Table 1; (6,14). In addition, preliminary tests were run with models that included methodological factors, specifically sequencing platform, sampling year, DNA extraction method, tissue type, and seed origin (S3 Table), as these have shown to influence fungal community composition in other studies (52).

**Table 1.** Description of predictors considered in models of fungal community and host health responses. Host species names in bold indicate host for which models of host and density and host and distance interaction were analyzed.

| **Predictor** | **Description** | **Levels** |
|---|---|---|
| Host | Species of seedling sampled for fungal community analysis | *A. saccharum* Marshall<br>*Carya alba* (Linnaeus)<br>*Fraxinus americana* L.<br>*Liquidambar styraciflua* L.<br>*Liriodendron tulipifera* L.<br>*Nyssa sylvatica* Marshall<br>*Pinus strobus* L.<br>*Q. falcata* Michx.<br>*Q. rubra* L. |
| Distance | Distance to closest conspecific adult | Far (>10 m)<br>Near (<10 m) |
| Density | Density of conspecific seedlings | High (all seedlings within a plot of the same species)<br>Low |
| Temperature | Refers to both air and soil temperature, if manipulated | Ambient (no manipulation)<br>Elevated (seedlings sampled within air and soil warming chambers) |
| Site | Six sampling sites were included in the analysis, with three sites each in Harvard Forest and Duke Forest. | Duke Forest Warming Site (DFWS)<br>Duke Forest Eno West (DFEW)<br>Duke Forest Blackwood (DFBW)<br>Harvard Forest Simes (HFST)<br>Harvard Forest Barre Woods (HFBW)<br>Harvard Forest Warming Site (HFWS) |
| Light | Light availability based on locations of open gaps at each site | Understory<br>Gap |
| Sequencing | Sequencing platform used for fungal community characterization | Roche 454 (R454)<br>Illumina MiSeq (MiSeq) |

Models testing the effects of the biotic and abiotic predictors (host species, light availability; temperature; site; distance to adult tree and diversity of neighboring seedlings; and sequencing platform, S4 Table) including the interactions between host and distance to conspecific adult (host*distance) and/or the interaction between host and diversity of neighboring seedlings (host*diversity) were run in R's *gjam* package version 2.5.2 (37). For the analysis in *gjam*, seedlings from 9 host species were included, corresponding to the species planted in the PSF plots, and for which host/density and host/diversity interactions could be analyzed (Table 1, S2 Table). Also, prior to the analysis, the fungal community data was filtered to include only fungal OTUs found in at least 10% of the studied samples, resulting in a total of 247 fungal OTUs from 521 samples. All models were run with *Acer saccharum* as the reference for the host variable and "low density" as reference for conspecific density. All models were run with Year as a covariate (S4 Fig; S4 Table). Models were evaluated based on their associated deviance information criterion (DIC) scores, and the model with the lowest DIC score was chosen as the best model (S4 Table) for further interpretation. The analysis with *gjam* resulted in the following outputs used for data interpretation: (A): Sensitivity analysis of response variables to individual predictors (and their interactions, Fig 1); (B): Predicted fungal OTUs and host health status responses to individual predictors (Figs 2, 3 and S5-S9 Figs), (C): Clustering of fungal OTUs and host health status based on similarity of responses to predictors (S10 Fig).

## Results

### *Data exploration and variable selection*

The analysis of 521 seedlings resulted in a total of 5,485,558 sequences representing 2,889 LSU OTUs. On average, 13,039 and 816 sequences per sample were recovered from the Illumina MiSeq and 454-pyrosequencing runs, respectively, representing 126 (± 78) and 40 (± 26) OTUs per sample, per sequencing platform (S5 and S6 Tables). The taxa recovered, and included in the analysis, belonged to the Kingdoms Fungi and Oomycota. The ten most abundant OTUs (Colletotrichum_1, Diaporthales_3, Sordariomycetes_186, Amphisphaeriaceae_6, Ceratobasidium_2, Neofabraea_3, Cladosporium_1, Neofusicoccum_2, Chalara_1, Helotiales_26) were found in both symptomatic and asymptomatic seedlings of most host species analyzed (S2 Fig). OTU richness (Chao1 index) recovered from seedlings at Duke Forest differed between host species and when recovered from seedlings at different densities of conspecific hosts (S7 Table, S3 Fig). In addition, significant interactions between host species and distance to conspecifics, and interactions between distance to conspecific adults and density of conspecific seedlings were observed for Shannon diversity estimates from Duke Forest seedlings. For seedlings collected in Harvard Forest, host species had a significant effect on diversity estimates (Shannon index), and significant interactions were observed for OTU richness between symptomatic status and conspecific density (S7 Table, S3 Fig).

GJAM was applied to determine the strengths of nine methodological predictors on fungal communities and host health status. First, sensitivity analysis of these predictors indicated that year had the strongest impact on the recovered fungi (S4 Fig), compared to

other sampling and sample processing parameters; therefore, year was included as a covariate in subsequent models tested during the model selection approach. Other predictors (sequencing method, DNA extraction method) with a high sensitivity covaried with year, so we included only year in subsequent models. The model with the best fit, based on DIC scores, included the interactions between the predictors host and density of conspecifics (density * host), the interaction between the predictors host and distance to a conspecific adult (distance * host), and the abiotic predictors temperature, site, and light (S4 Table).

***Community level responses to the model with the best fit***

We used the best fitting model (S4 Table) to explain the joint responses of fungal species and health status of the studied seedlings. The contributions of the individual predictors included in the selected model was compared via sensitivity analysis (Fig 1), where high sensitivity score indicates larger contribution of a predictor to the joint response of the 247 fungal taxa selected for analysis. Individually, forest site and host species were stronger predictors compared to other abiotic parameters (Fig 1). However, regardless of host species, the interaction between host species and density of conspecific seedlings and/or host species and distance to a conspecific adult was almost always a stronger predictor of fungal community and host health than host identity alone (Fig 1).

**Fig 1.** Sensitivity of fungal OTUs and host health status to biotic (host, distance and density), abiotic (temperature, site, light) and sequencing as predictors. Predictors are colored and labeled according to: Distance (blue) F=far, N=near; Density (green) HIGH=high density of conspecifics, LOW=low density of conspecifics; Light (yellow) G= gap, U= understory; TempTreat (red) A=ambient temperature, E=elevated temperature; Site (orange): DFEW, DFBW, DFWS=Duke Forest Eno West, Blackwood and Warming Site, respectively; HFBW, HFST, HFWS=Harvard Forest Barre Woods, Simes Tract and Warming Site, respectively; Sequencing (gray) = MiSeq and Roche454 Sequencing platforms; and hosts (purple, Table 1).

Interactions between host identity and density of conspecific seedlings (host*density), or host identity and distance to conspecifics (host*distance) also impacted the presence or absence of individual fungal taxa. GJAM evaluated response coefficients for all fungal taxa-predictor combinations, and Fig 2 summarizes individual fungal taxa responses to host*density and host*distance interactions for the plant hosts *L. styraciflua* and *L. tulipifera*. Results for other interactions are shown in S5 Fig and S6 Fig. Individual fungal taxa were identified as preferentially recovered under each interaction when the response coefficient was greater than zero, or preferentially absent under each interaction when the response coefficient was less than zero. For instance, six fungal taxa were preferentially recovered (coefficient >0) from the host *L. tulipifera* when grown near a conspecific adult (Fig 2B). In addition, five of these taxa were different from the six fungal taxa preferentially recovered from the host *L. styraciflua* when grown near a conspecific adult (Fig 2D). Further, the fungus classified as Colletotrichum1 was preferentially recovered from both *L. styraciflua* and *L. tulipifera* when grown near

conspecific adults, while the taxon Helotiales6 was preferentially recovered from both hosts when grown in areas of low density to conspecifics.

**Fig 2.** Posterior distributions of fungal OTUs responses to host species *L.tulipifera* (A,B) and *L. styraciflua* (C,D) and their interaction with density of conspecific seedlings (A,C) or distance to a conspecific adult (B,D). Taxa with credible intervals different than zero are shown. Results from other hosts and their interactions with density and distance are shown in S5 and S6 Figures, respectively; and host effect only on S8 Fig.

The amount of individual fungal taxa responding to different predictors showed some patterns. Collectively, many fungal taxa responded (either positively or negatively) to the interactions between host and distance or host and density (Table 2, S5-S6 Figs, S8 Table). More responses were observed for host*distance interactions (107) than host *density (90). In some cases, the number of fungal taxa responding to distance or density were asymmetric. For example, four times more fungal taxa responded to far distance from conspecific adults in *F. americana*, and five times more responded to far distance in *N. sylvatica*. Similarly, five times more taxa responded to low density when the host was *C. alba* and three times more taxa responded to high density when the host was *Q. rubra*.

**Table 2.** Number of fungal taxa responses to plant host identity, the interactions between host identity with the density of conspecific seedlings, and the interaction of host identity with distance to conspecific adults.

| | **ACSA** | **CATO** | **FRAM** | **LIST** | **LITU** | **NYSY** | **PIST** | **QUFA** | **QURU** |
|---|---|---|---|---|---|---|---|---|---|
| Positive | 12 | 9 | 8 | 9 | 9 | 9 | 7 | 9 | 6 |
| Negative | 10 | 4 | 10 | 7 | 7 | 8 | 6 | 5 | 3 |
| Host*density | | | | | | | | | |
| High | Ref | 1 | 9 | 9 | 6 | 6 | 6 | 4 | 3 |
| Low | Ref | 5 | 10 | 7 | 7 | 8 | 6 | 2 | 1 |
| Host*distance | | | | | | | | | |
| Far | Ref | 6 | 12 | 9 | 8 | 20 | 7 | 2 | 6 |
| Near | Ref | 5 | 3 | 5 | 6 | 4 | 4 | 5 | 5 |

Similar to host and conspecific density and distance interactions, different groups of fungal taxa were preferentially recovered from seedlings sampled at different sites, and also within sites in locations that differed in light availability and warming treatments (Fig 3, S7 Fig). 38 and 25 taxa were preferentially recovered from Duke Forest and Harvard Forest sites, respectively. A subset of 22 or 24 taxa significantly responded to experimental warming and light availability, respectively. For example, one fungal taxon, Chalara1, was preferentially recovered from seedlings grown under low light (understory) and ambient temperature; whereas the taxon Melanopsammella1 was preferentially recovered from seedlings grown in the understory and under elevated

temperature. Overall, eleven taxa were preferentially recovered under warmer temperatures, and eleven preferentially recovered under ambient (Fig 3A). Of the 22 taxa who responded to temperature, 18 also responded (either positively or negatively) to distance or density in at least one host species, indicating a potential interaction between temperature and distance or density. Site-specific patterns were also observed; for example, Colletotrichum1 was preferentially recovered from Harvard Forest seedlings, whereas Colletotrichum4 from Duke Forest seedlings.

**Fig 3.** Posterior distributions of fungal OTUs responses to environmental predictors, including light availability (A), elevated temperature (B), and site (C and D). . Taxa with credible intervals different than zero are shown. Light availability was controlled based on open gaps in the canopy (vs. understory). A subset of seedlings were exposed to elevated temperatures at Warming experiments at each forest (vs. ambient temperature). Two examples for site effect are shown for Harvard Forest Simes Tract and Duke Forest Eno West. Additional sites are shown in S7 Fig.

Overall, from the 247 fungal taxa included in the analysis, 109 taxa showed preferential recovery when seedlings were grown under at least one of the studied predictors and/or their interactions (Fig 4), with a subset responding to multiple predictors. Six fungal taxa (Agaricales35, Russulaceae1, Colletotrichum1, Pleosporales18, Ceratobasidium2 and Diaporthaceae1) recovery were modeled at least half of the time (17 out of the possible 35 combinations; S9 Table). From these taxa, Colletotrichum1 and Diaporthaceae1 responded to eight and four of the host*distance combinations respectively, and Diaporthaceae1 to three of the host*density combinations. However, Colletotrichum1

was preferentially recovered from the various hosts at the near distance; whereas Diaporthaceae1 was preferentially recovered from three hosts at the distance farther from the conspecific adult (*L.styraciflua*, *L. tulipifera* and *N. sylvatica*) and one at closer distance (*Q. falcata*). Further, Diaporthaceae1 was recovered from seedlings of *F. americana*, *L. tulipifera* and *N. sylvatica* when grown under low conspecific density.

**Fig 4.** Number of significant responses to predictors per taxa, based on posterior distributions being $\neq 0$ for the studied predictors and their combination. The total number of responses per individual taxa is summarized, where the y-axis indicates total number of taxa per individual response number (x-axis).

***Structural analysis of community level response***

To determine if different taxa show similar patterns of responses to predictors, GJAM estimates the correlation between individual taxa responses to predictors and clusters taxa based on both these correlations and the posterior distributions of each taxa/predictor combinations. These taxa clustering is shown in the left panel of S10 Fig and the taxa clusters are summarized in S10 Table. Analysis of the structure of responses of the fungal communities to the tested predictors (Table 1) revealed four main clusters of taxa. The strength of correlation between fungal taxa and their posterior distributions is shaded as a heat map, from blue being strong negative to red being strong positive response. Four clusters (labeled as 1-4) summarize commonalities in response to predictors in taxa responses to the model (depicted as red triangles in the left panel of Figure S10, labeled 1-4). Each of the four clusters, contains 139, 67, 12, 29 fungal taxa, respectively for

clusters one to four, respectively. The response variable host health status, clusters in group 1.

The impact of the predictors on particular fungal taxa is shown in the right panel of S10 Fig, where the heat map color indicates contribution of the predictor to the individual fungal taxon response (strong positive red to strong negative blue). Individual predictors clustered based on their contributions to the model (cluster shown in the top right panel). For example, the interaction between the host *Q. falcata* and low density of conspecifics separates from the rest of predictors. The rest of the predictors cluster based on distance and host species, density and host species interactions, or abiotic parameters. No consistent patterns are observed in the clustering of predictors among each taxa clusters (right panel, S10 Fig), indicating that while there is co-occurrence of certain taxa, it is not necessarily related to the predictors tested.

**Discussion**

In this study, we tested the hypothesis that fungal communities in tree seedlings are modulated by host identity, conspecific seedling density, distance to conspecific adults, and several abiotic covariates, most notably experimental warming. We found that each tree species harbors a distinct community of fungi, but the structure of the fungal community is modulated by characteristics in ways that differ between host plants. Interactions between host identity and density of conspecifics, or host identity and distance to conspecific adults, exerted stronger influence over fungal communities than

environmental variables that included warming, light availability, and forest site, and even host identity alone (Fig 1). Although fungal taxa are certainly generalists as gauged by the traditional view of numbers of hosts they infect (S2 Fig), we found that the host by conspecific seedling density interaction and host by distance to conspecific adult interaction is fungal-taxon specific (Fig 2, S5-S6 Fig). This suggests that fungal host specialization occurred both at the individual taxon (OTU) level and the fungal community as a whole.

Unique sets of fungal taxa responded to each biotic (i.e. distance to conspecific adults and density of conspecifics) and environmental (i.e. warming, light, site) variable. However, the responses of fungal OTUs to hosts depended on the presence and location of conspecific individuals (Fig 2 and S5-S6 Figs). This result may explain previous observations of either distance or density dependent responses (but not both) in different forests (12,53,54). Distance and density responses are not mutually exclusive, but instead both can lead to Janzen-Connell-like responses from different members of the fungal community (55). Most taxa responded to a limited number of predictors, but a small subset responded to more than ten (Fig 4).

Taxon-specific responses to host, site and environment (e.g. warming, light) (Figs 2-3 and S7-8 Figs) were consistent with previous studies showing that seedling survival was differentially affected by combinations of generalist fungi, depending on soil moisture (6). Host-associated microbial communities that are modulated by environment have been described in other plant species. For instance, host genotype effects of the

herbaceous plant *Boechera stricta* on the leaf bacterial microbiome was modulated by site. Host genotype by site interactions could then be used to predict the abundance of bacterial taxa in *Boechera stricta* (56). The set of fungal taxa that responded to different combinations of hosts and predictors set the stage for subsequent experimental studies that verify the role of different fungal taxa in seedling survival. Notably for future studies in these ecosystems, this work highlighted a subset of fungal taxa that responded, positively and negatively, to elevated temperatures consistent with a warming world (Fig 3A). The majority of the species who responded to temperature (18/22, 81%) also had some kind of host-specific differential response to distance and/or density, indicating a potential interaction between the impacts of conspecific negative density dependence and warming that could be explored further. Large-scale experimental studies exploring the interactions between individual fungi and hosts (5) can be challenging to design given the many potential combinations; modeling approaches like this prune the list of taxa-host combinations that sets the stage for future experimental work. This is particularly important in studies of warming effects on plant-soil feedbacks, which are currently quite limited.

Communities of fungi did not respond equally to all predictors. Analysis of the structure of responses of taxa to predictors show clusters of fungi that differ in the strength to which they respond to the analyzed predictors (S10 Fig and S8-S10 Tables). However, based on this analysis, it is not evident to which specific predictors these clusters are responding, or alternatively, the clustering is influenced by non-measured variables. In previous research (57–59), fungal communities are often treated uniformly, based on

richness or evenness estimates or ordination axes as a dimension reduction strategy; or experimentally, through comparisons of comparing fungicide vs. non-fungicide treatment effect. Alternatively, specific host-pathogen interactions tend to be studied as a single interaction at a time. Instead, different members (or group of members) of a large and diverse community are responding differently to host and environmental factors collectively.

The application of GJAM models to the fungal sequencing survey presented in this work allowed an exhaustive analysis of the fungal communities to identify specialization of individual taxa and community. Individual taxa do not necessarily follow patterns of predicted functionality based on fungal taxonomy assignment and prior knowledge of fungal lifestyle, with the caveat that limited information on functionality is available given the taxonomy resolution used in this work. Changes in fungal community composition in response to conspecific density and distance to conspecific adults is observed for plant pathogens, beneficial symbionts and saprotrophs. While CNDD interactions have been reported previously for mycorrhizal host associations, we note that these interactions are evaluated for seedlings > 1 year old, and that to our knowledge mycorrhizal CNDD interactions have not been noted for tree seedlings less than 1 year old (24,60). For this reason, we did not evaluate CNDD within the context of host mycorrhizal status for this study. Further, the unequal gemination between AM and EM tree species in this study confounded potential comparisons. However, hypotheses on shared functions could be developed for clusters of taxa with similar responses to predictors. For instance, subsets of the analyzed fungal OTUs share responses to the

model predictors; and contrasting responses to predictors are observed by individual taxa. For example, though both Colletotrichum1 and Diaporthacea1 were recovered from several hosts and respond to multiple host and density interactions, Colletotrichum1 was preferentially recovered from distances near to a conspecific host; whereas Diaporthacea1 from further distances, as well as under low density of conspecifics. Determining the functions of the recovered fungal taxa, however, remains dependent on better taxonomic resolution, along with additional functional data from methods such as enzyme assays, genomics, and pathogenicity analysis. Previous studies that had identified pathogen drivers of plant community dynamics focused on a combination of culture-based surveys (e.g. 6,16,61,62), amplicon based surveys (63–68) or fungicide disturbance treatments (69). Even though cultured isolates provide an advantage when testing hypotheses of function, extensive surveys and factorial interactions combine to make it prohibitive to carry out these tests using diverse hosts and environmental conditions. Studies using a molecular approach have linked increased putative pathogen frequency to conspecific distance or density (19,21,67,70), but have not considered variation due to environmental factors.

By employing GJAM, this study was able to directly identify the responses of the fungal community and individual fungal OTUs to biotic and environmental variables, as well as interactions relevant to negative conspecific density dependence. It also highlights groups of fungi that share similar responses to predictors, and potentially functionality. The fact that different collections of fungi are associated with each tree, but that these fungi are responding to different suites of predictors, also suggests that effective specialization is

mediated by interactions between biotic and abiotic factors. These differences in fungal community composition and individual taxa responses to host and environment interactions can explain niche partitioning effects on seedling survival (71) from the perspective of the fungal communities. Fungal taxa responding specifically to elevated temperature, both positive and negative, should be explored further to better understand the potential changes in plant-fungal interactions under climate change. The data presented here supports the hypothesis of mechanisms through which effective specialization allow generalist pathogens to mediate plant community dynamics (14).

**Acknowledgements**

For field and lab assistance we thank B. Viera, B. Roper, B. Spakes-Richter, A. Barker Plotkin, A. Lewis, R. DeMatte, M. Campbell and M. Pasay. We thank the Environmental Metagenomics course at Sarah Lawrence College for their comments to this manuscript. This work was funded by the National Science Foundation grant NSF-DEB-0955904.

**Data Availability Statement**

The sequence and associated metadata that supports the findings of this study were submitted to Qiita online repository. Additional data are available from the corresponding author upon reasonable request.

**Author contributions**

MH Hersh, R Vilgalys and JS Clark conceptualized the forest experiments. MH Hersh, MS Benitez Ponce and L Becker administered and performed the experiments. MS Benitez Ponce performed all analyses and data curation. MS Benitez Ponce, MH Hersh, L Becker, wrote/reviewed the original article and contributed to data visualization. R Vilgalys and JS Clark supervised the work, acquired funding and reviewed/edited the manuscript.

## References


1. Terborgh J. At 50, Janzen–Connell Has Come of Age. BioScience. 2020 Dec;70(12):1082–92.
2. Stump SM, Comita LS. Differences among species in seed dispersal and conspecific neighbor effects can interact to influence coexistence. Theor Ecol. 2020 Dec 1;13(4):551–81.
3. Janzen DH. Herbivores and the number of tree species in tropical forests. Am Nat. 1970;104(940):501–28.
4. Connell JH. On the role of natural enemies in preventing competitive exclusion in some marine animals and in rain forest trees. Dyn Popul. 1971;298:312.
5. Spear ER, Broders KD. Host-generalist fungal pathogens of seedlings may maintain forest diversity via host-specific impacts and differential susceptibility among tree species. New Phytol. 2021 Jul;231(1):460–74.
6. Hersh MH, Vilgalys R, Clark JS. Evaluating the impacts of multiple generalist fungal pathogens on temperate tree seedling survival. Ecology. 2012;93(3):511–20.
7. Bonito G, Reynolds H, Robeson II MS, Nelson J, Hodkinson BP, Tuskan G, et al. Plant host and soil origin influence fungal and bacterial assemblages in the roots of woody plants. Mol Ecol. 2014;23(13):3356–70.

8. Delgado-Baquerizo M, Guerra CA, Cano-Díaz C, Egidi E, Wang JT, Eisenhauer N, et al. The proportion of soil-borne pathogens increases with warming at the global scale. Nat Clim Change. 2020 Jun;10(6):550–4.
9. Liu Y, He F. Warming intensifies soil pathogen negative feedback on a temperate tree. New Phytol. 2021 Sep;231(6):2297–307.
10. Comita LS, Queenborough SA, Murphy SJ, Eck JL, Xu K, Krishnadas M, et al. Testing predictions of the Janzen–Connell hypothesis: a meta-analysis of experimental evidence for distance-and density-dependent seed and seedling survival. J Ecol. 2014;102(4):845–56.
11. Zhu K, Woodall CW, Monteiro JVD, Clark JS. Prevalence and strength of density-dependent tree recruitment. Ecology. 2015 Sep;96(9):2319–27.
12. Johnson DJ, Beaulieu WT, Bever JD, Clay K. Conspecific negative density dependence and forest diversity. Science. 2012;336(6083):904–7.
13. Brown AJ, White PS, Peet RK. Environmental context alters the magnitude of conspecific negative density dependence in a temperate forest. Ecosphere [Internet]. 2021 Mar [cited 2023 Jul 17];12(3). Available from: https://onlinelibrary.wiley.com/doi/10.1002/ecs2.3406
14. Benítez MS, Hersh MH, Vilgalys R, Clark JS. Pathogen regulation of plant diversity via effective specialization. Trends Ecol Evol. 2013 Dec 1;28(12):705–11.
15. Bever JD, Mangan S, Alexander HM. Maintenance of plant species diversity by pathogens. Annu Rev Ecol Evol Syst. 2015 Dec 2;46(1):305–25.

16. Yamazaki M, Iwamoto S, Seiwa K. Distance- and density-dependent seedling mortality caused by several diseases in eight tree species co-occurring in a temperate forest. Plant Ecol. 2009;201(1):181–96.
17. Spear ER, Coley PD, Kursar TA. Do pathogens limit the distributions of tropical trees across a rainfall gradient? J Ecol. 2015 Jan;103(1):165–74.
18. Schroeder JW, Martin JT, Angulo DF, Barbosa JM, Perea R, Arias-Del Razo I, et al. Community composition and diversity of Neotropical root-associated fungi in common and rare trees. Biotropica. 2018 Jul 1;50(4):694–703.
19. Chen L, Swenson NG, Ji N, Mi X, Ren H, Guo L, et al. Differential soil fungus accumulation and density dependence of trees in a subtropical forest. Science. 2019 Oct 4;366(6461):124–8.
20. Chen Y, Jia P, Cadotte MW, Wang P, Liu X, Qi Y, et al. Rare and phylogenetically distinct plant species exhibit less diverse root-associated pathogen communities. Figshare. 2018 04;
21. Mommer L, Cotton TEA, Raaijmakers JM, Termorshuizen AJ, van Ruijven J, Hendriks M, et al. Lost in diversity: the interactions between soil-borne fungi, biodiversity and plant productivity. New Phytol. 2018 Apr 1;218(2):542–53.
22. Chung YA, Jumpponen A, Rudgers JA. Divergence in Diversity and Composition of Root-Associated Fungi Between Greenhouse and Field Studies in a Semiarid Grassland. Microb Ecol. 2019 Jul 1;78(1):122–35.
23. Bennett JA, Maherali H, Reinhart KO, Lekberg Y, Hart MM, Klironomos J. Plant-soil feedbacks and mycorrhizal type influence temperate forest population dynamics. Science. 2017 Jan 13;355(6321):181–4.

24. Jiang F, Zhu K, Cadotte MW, Jin G. Tree mycorrhizal type mediates the strength of negative density dependence in temperate forests. Beckman N, editor. J Ecol. 2020 Nov;108(6):2601–10.
25. Jiang F, Lutz JA, Guo Q, Hao Z, Wang X, Gilbert GS, et al. Mycorrhizal type influences plant density dependence and species richness across 15 temperate forests. Ecology [Internet]. 2021 Mar [cited 2022 Jul 27];102(3). Available from: https://onlinelibrary.wiley.com/doi/10.1002/ecy.3259
26. Augspurger CK, Kelly CK. Pathogen Mortality of Tropical Tree Seedlings - Experimental Studies of the Effects of Dispersal Distance, Seedling Density, and Light Conditions. Oecologia. 1984;61(2):211–7.
27. McCarthy-Neumann S, Ibanez I. Plant-soil feedback links negative distance dependence and light gradient partitioning during seedling establishment. Ecology. 2013 Apr;94(4):780–6.
28. Kaisermann A, de Vries FT, Griffiths RI, Bardgett RD. Legacy effects of drought on plant-soil feedbacks and plant-plant interactions. New Phytol. 2017 Sep;215(4):1413–24.
29. Milici VR, Dalui D, Mickley JG, Bagchi R. Responses of plant–pathogen interactions to precipitation: Implications for tropical tree richness in a changing world. Fridley J, editor. J Ecol. 2020 Sep;108(5):1800–9.
30. Kobe RK. Light gradient partitioning among tropical tree species through differential seedling mortality and growth. Ecology. 1999 Jan 1;80(1):187–201.

31. Battaglia LL, Foré SA, Sharitz RR. Seedling emergence, survival and size in relation to light and water availability in two bottomland hardwood species. J Ecol. 2000 Dec 1;88(6):1041–50.
32. Caballol M, Méndez-Cartín AL, Serradó F, De Cáceres M, Coll L, Oliva J. Disease in regenerating pine forests linked to temperature and pathogen spillover from the canopy. J Ecol. 2022 Nov;110(11):2661–72.
33. Liu X, Ma Z, Cadotte MW, Chen F, He JS, Zhou S. Warming affects foliar fungal diseases more than precipitation in a Tibetan alpine meadow. New Phytol. 2019 Feb;221(3):1574–84.
34. Liu Y, He F. Warming shifts soil microbial communities and tropical tree seedling mortality. Ecology. 2022 Dec;103(12):e3810.
35. Huang K, Kardol P, Yan X, Luo X, Guo H. Plant–soil biota interactions explain shifts in plant community composition under global change. Funct Ecol. 2021 Dec;35(12):2778–88.
36. Gundale MJ, Kardol P. Multi-dimensionality as a path forward in plant-soil feedback research. J Ecol. 2021 Oct;109(10):3446–65.
37. Clark JS, Nemergut D, Seyednasrollah B, Turner PJ, Zhang S. Generalized joint attribute modeling for biodiversity analysis: median-zero, multivariate, multifarious data. Ecol Monogr. 2017 Feb;87(1):34–56.
38. Clark JS, Salk C, Melillo J, Mohan J. Tree phenology responses to winter chilling, spring warming, at north and south range limits. Funct Ecol. 2014 Dec;28(6):1344–55.

39. Gardes M, Bruns TD. ITS primers with enhanced specificity for basidiomycetes - application to the identification of mycorrhizae and rusts. Mol Ecol. 1993;2(2):113–8.
40. McNickle GG, Cahill JF, Deyholos MK. A PCR-based method for the identification of the roots of 10 co-occurring grassland species in mesocosm experiments. Botany. 2008;86(5):485–90.
41. Johansen RB, Johnston P, Mieczkowski P, Perry GL, Robeson MS, Burns BR, et al. A native and an invasive dune grass share similar, patchily distributed, root-associated fungal communities. Fungal Ecol. 2016;23:141–55.
42. Bonito G, Reynolds H, Robeson MS, Nelson J, Hodkinson BP, Tuskan G, et al. Plant host and soil origin influence fungal and bacterial assemblages in the roots of woody plants. Mol Ecol. 2014;23(13):3356–70.
43. Gonzalez A, Navas-Molina JA, Kosciolek T, McDonald D, Vázquez-Baeza Y, Ackermann G, et al. Qiita: rapid, web-enabled microbiome meta-analysis. Nat Methods. 2018 Oct 1;15(10):796–8.
44. Caporaso JG, Kuczynski J, Stombaugh J, Bittinger K, Bushman FD, Costello EK, et al. QIIME allows analysis of high-throughput community sequencing data. Nat Methods. 2010 May;7(5):335–6.
45. Bragg L, Stone G, Imelfort M, Hugenholtz P, Tyson GW. Fast, accurate error-correction of amplicon pyrosequences using Acacia. Nat Meth. 2012;9(5):425–6.
46. Edgar RC. UPARSE: highly accurate OTU sequences from microbial amplicon reads. Nat Methods. 2013 Oct;10(10):996–8.

47. Martin M. Cutadapt removes adapter sequences from high-throughput sequencing reads. EMBnet.journal. 2011 May 2;17(1):10.
48. McMurdie PJ, Holmes S. phyloseq: An R Package for Reproducible Interactive Analysis and Graphics of Microbiome Census Data. Watson M, editor. PLoS ONE. 2013 Apr 22;8(4):e61217.
49. R Core Team. R: A language and environment for statistical computing. R Foundation for Statistical Computing, Vienna, Austria. [Internet]. 2018. Available from: https://www.R-project.org
50. Gloor GB, Macklaim JM, Pawlowsky-Glahn V, Egozcue JJ. Microbiome Datasets Are Compositional: And This Is Not Optional. Front Microbiol [Internet]. 2017 [cited 2023 Jun 24];8. Available from: https://www.frontiersin.org/articles/10.3389/fmicb.2017.02224
51. Kaul A, Mandal S, Davidov O, Peddada SD. Analysis of Microbiome Data in the Presence of Excess Zeros. Front Microbiol [Internet]. 2017 [cited 2023 Jun 24];8. Available from: https://www.frontiersin.org/articles/10.3389/fmicb.2017.02114
52. Song Z, Schlatter D, Gohl DM, Kinkel LL. Run-to-Run Sequencing Variation Can Introduce Taxon-Specific Bias in the Evaluation of Fungal Microbiomes. Phytobiomes J. 2018 Jan 1;2(3):165–70.
53. Boudreau S, Lawes MJ. Density- and Distance-Dependent Seedling Survival in a Ballistically Dispersed Subtropical Tree Species Philenoptera sutherlandii. J Trop Ecol. 2008;24(1):1–8.
54. Packer A, Clay K. Soil pathogens and spatial patterns of seedling mortality in a temperate tree. Nature. 2000;404(6775):278–81.

55. Terborgh J. Enemies Maintain Hyperdiverse Tropical Forests. Am Nat. 2012;179(3):303–14.

56. Wagner MR, Lundberg DS, del Rio TG, Tringe SG, Dangl JL, Mitchell-Olds T. Host genotype and age shape the leaf and root microbiomes of a wild perennial plant. Nat Commun. 2016 Jul 12;7:12151.

57. Lin HA, Mideros SX. The Effect of Septoria glycines and Fungicide Application on the Soybean Phyllosphere Mycobiome. Phytobiomes J. 2022 Jul 29;PBIOMES-12-21-0075-R.

58. Rojas EC, Sapkota R, Jensen B, Jørgensen HJL, Henriksson T, Jørgensen LN, et al. Fusarium Head Blight Modifies Fungal Endophytic Communities During Infection of Wheat Spikes. Microb Ecol. 2020 Feb;79(2):397–408.

59. Simonin M, Briand M, Chesneau G, Rochefort A, Marais C, Sarniguet A, et al. Seed microbiota revealed by a large-scale meta-analysis including 50 plant species. New Phytol. 2022;234(4):1448–63.

60. Qin J, Geng Y, Li X, Zhang C, Zhao X, von Gadow K. Mycorrhizal type and soil pathogenic fungi mediate tree survival and density dependence in a temperate forest. For Ecol Manag. 2021 Sep 15;496:119459.

61. Packer A, Clay K. Soil pathogens and spatial patterns of seedling mortality in a temperate tree. Nature. 2000;404(6775):278–81.

62. Sarmiento C, Zalamea PC, Dalling JW, Davis AS, Stump SM, U’Ren JM, et al. Soilborne fungi have host affinity and host-specific effects on seed germination and survival in a lowland tropical forest. Proc Natl Acad Sci U S A. 2017 Oct 24;114(43):11458–63.

63. Liang M, Liu X, Gilbert GS, Zheng Y, Luo S, Huang F, et al. Adult trees cause density-dependent mortality in conspecific seedlings by regulating the frequency of pathogenic soil fungi. Ecol Lett. 2016 Dec;19(12):1448–56.
64. Chen L, Swenson NG, Ji N, Mi X, Ren H, Guo L, et al. Differential soil fungus accumulation and density dependence of trees in a subtropical forest. Science. 2019 Oct 4;366(6461):124.
65. Mommer L, Cotton TEA, Raaijmakers JM, Termorshuizen AJ, van Ruijven J, Hendriks M, et al. Lost in diversity: the interactions between soil-borne fungi, biodiversity and plant productivity. New Phytol. 2018 Apr 1;218(2):542–53.
66. Schroeder JW, Martin JT, Angulo DF, Barbosa JM, Perea R, Arias-Del Razo I, et al. Community composition and diversity of Neotropical root-associated fungi in common and rare trees. Biotropica. 2018 Jul 1;50(4):694–703.
67. Semchenko M, Leff JW, Lozano YM, Saar S, Davison J, Wilkinson A, et al. Fungal diversity regulates plant-soil feedbacks in temperate grassland. Sci Adv. 2018 Nov;4(11):eaau4578.
68. Chen Y, Jia P, Cadotte MW, Wang P, Liu X, Qi Y, et al. Rare and phylogenetically distinct plant species exhibit less diverse root-associated pathogen communities. Thrall P, editor. J Ecol. 2019 May;107(3):1226–37.
69. Bagchi R, Swinfield T, Gallery RE, Lewis OT, Gripenberg S, Narayan L, et al. Testing the Janzen-Connell mechanism: pathogens cause overcompensating density dependence in a tropical tree. Ecol Lett. 2010;13(10):1262–9.

70. Liang M, Liu X, Gilbert GS, Zheng Y, Luo S, Huang F, et al. Adult trees cause density-dependent mortality in conspecific seedlings by regulating the frequency of pathogenic soil fungi. Ecol Lett. 2016 Dec;19(12):1448–56.
71. Johnson DJ, Condit R, Hubbell SP, Comita LS. Abiotic niche partitioning and negative density dependence drive tree seedling survival in a tropical forest. Proc R Soc B. 2017;284(1869):20172210.

Figure 1

Click here to access/download;Figure;Figure1.sensitivity.tif

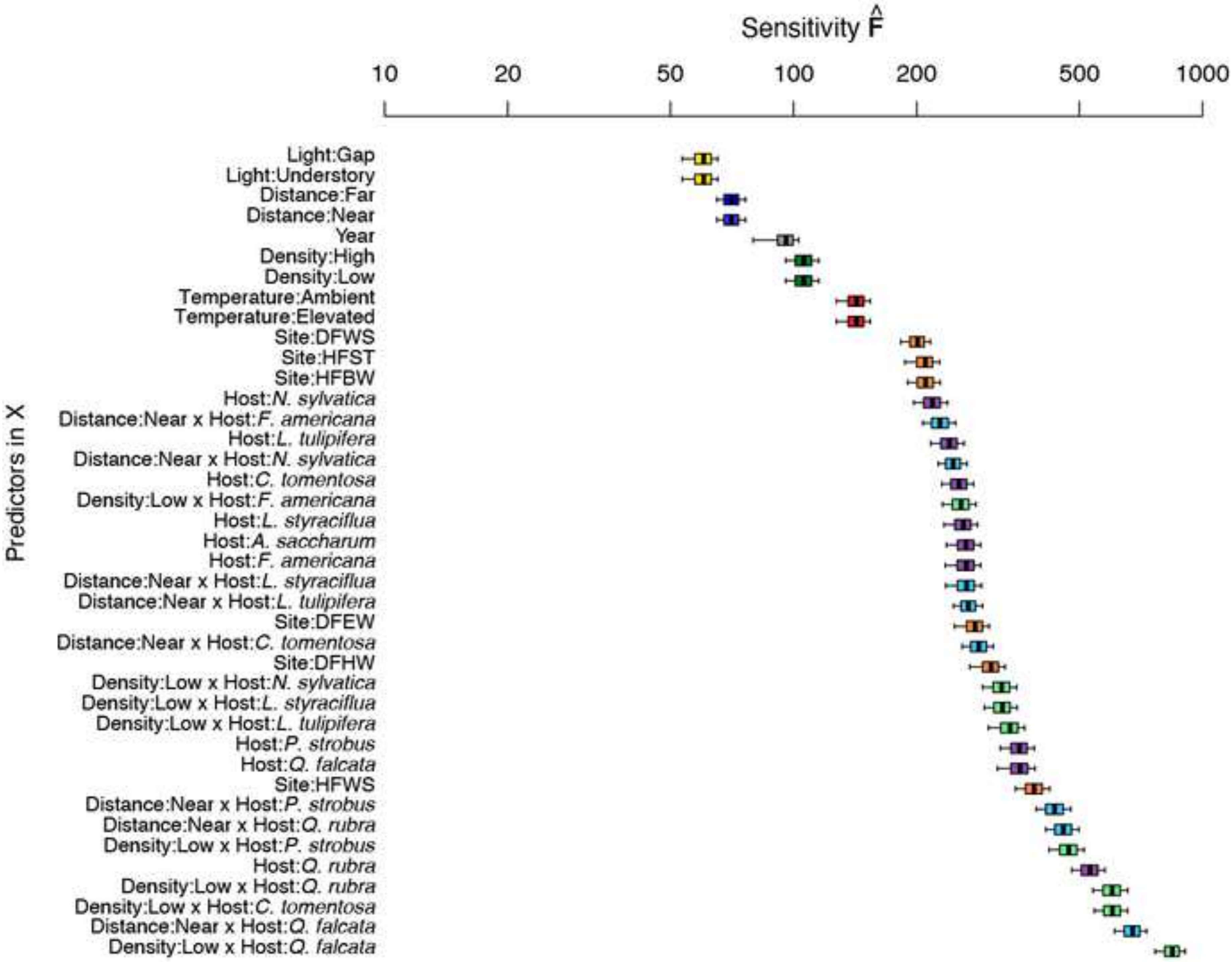

Figure 2

Click here to access/download;Figure;Figure2.biotic4panel.revised.tif

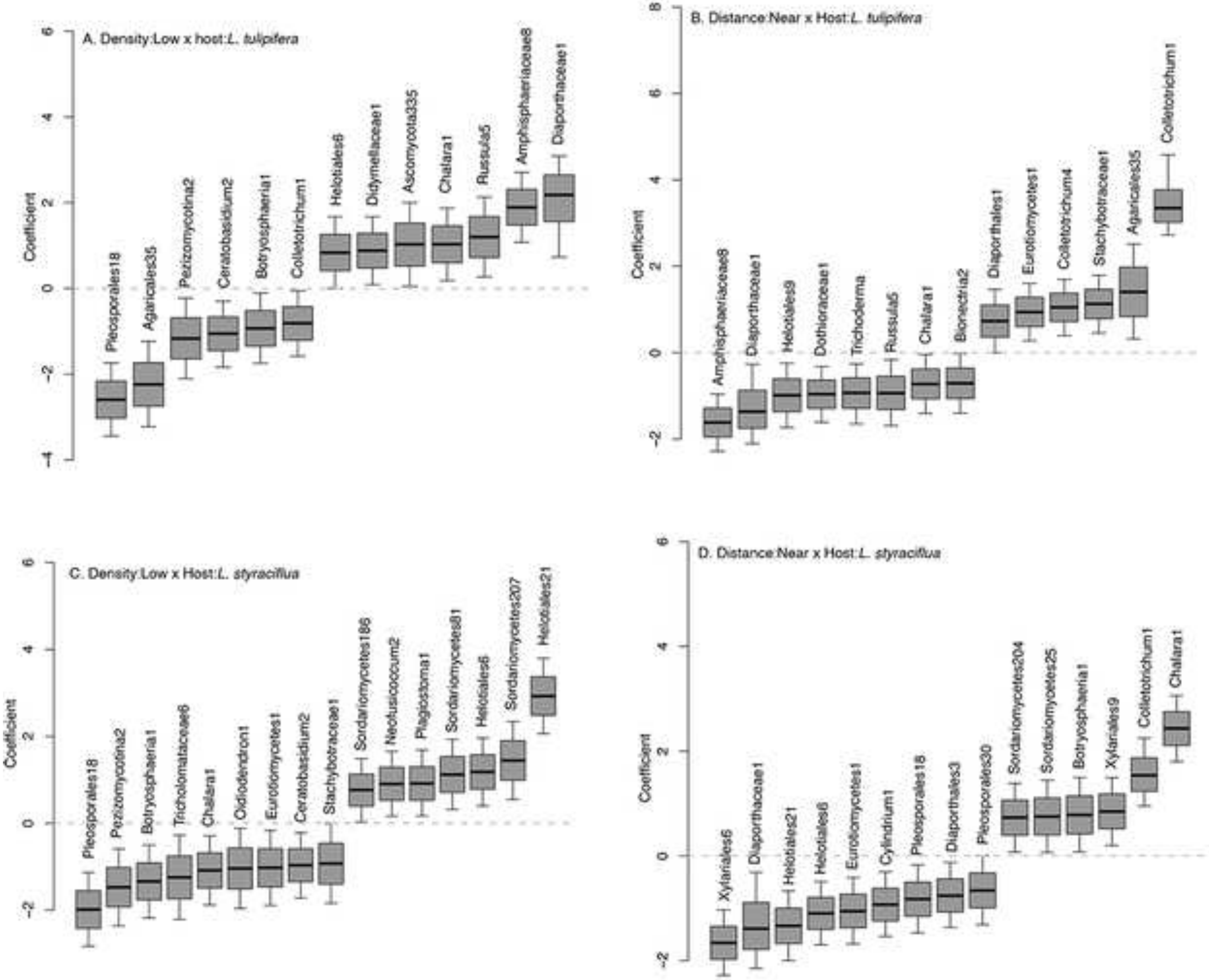

Figure 3 Click here to access/download;Figure;Figure3.Abiotic.FourPanel.tif

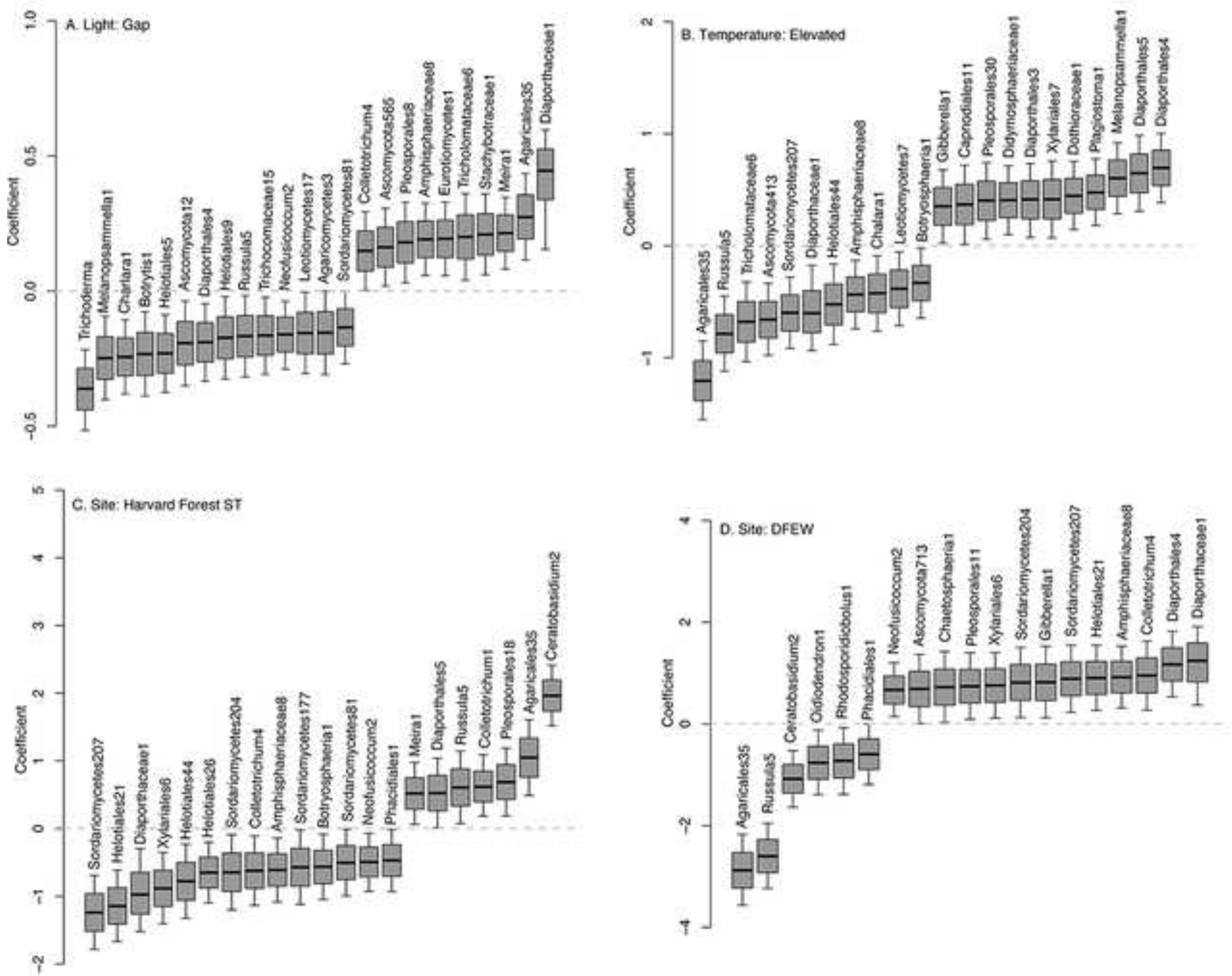

Figure 4

Click here to access/download;Figure;Figure4_Taxa_responses.tiff

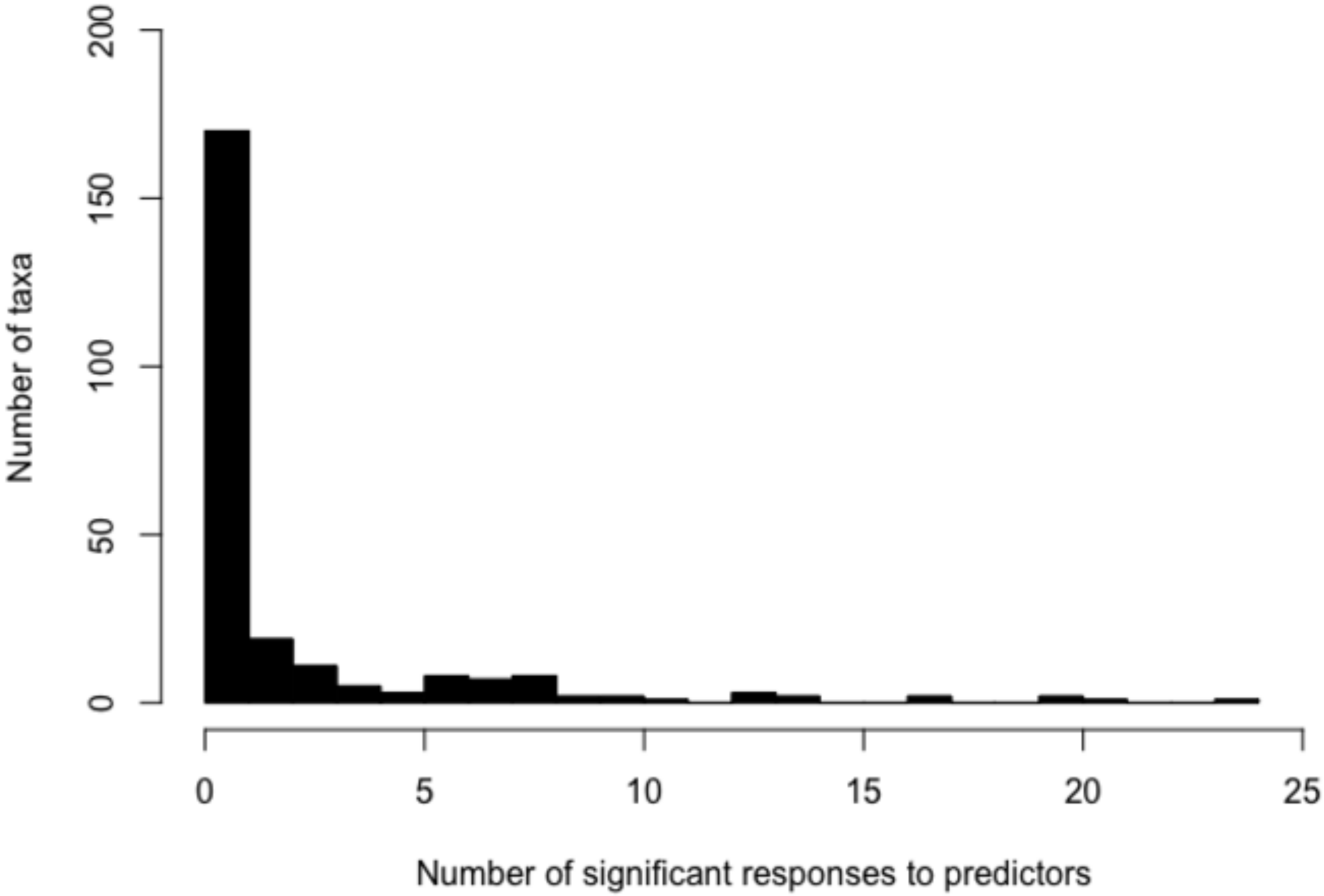

Click here to access/download
**Supporting Information**
Benitez_etal_SuppMethods_7-24.pdf

Supporting Figures

Click here to access/download
**Supporting Information**
Benitez_etal_SuppFigures_7-24.pdf

Supporting Tables

Click here to access/download
**Supporting Information**
Benitez_etal_SuppTables_7-24.pdf

Supporting Table 9

Click here to access/download
**Supporting Information**
Benitez_etal_SuppTable9.xlsx
